# Measuring health science research and development in Africa: mapping the available data

**DOI:** 10.1186/s12961-021-00778-y

**Published:** 2021-12-11

**Authors:** Clare Wenham, Olivier Wouters, Catherine Jones, Pamela A. Juma, Rhona M. Mijumbi-Deve, Joëlle L. Sobngwi-Tambekou, Justin Parkhurst

**Affiliations:** 1 Department of Health Policy, LSE, Houghton Street, London, WC2A 2AE United Kingdom; 2 LSE Health, LSE, Houghton Street, London, WC2A 2AE United Kingdom; 3 The Center for Rapid Evidence Synthesis, College of Health Sciences, Makerere University, Kampala, Uganda; 4 Africa Center for Evidence, University of Johannesburg, Johannesburg, South Africa; 5 RSD Institute (Recherche-Santé & Développement), Yaounde, Cameroon

**Keywords:** Health sciences research, Africa, Indicators, Capacity

## Abstract

**Background:**

In recent years there have been calls to strengthen health sciences research capacity in African countries. This capacity can contribute to improvements in health, social welfare and poverty reduction through domestic application of research findings; it is increasingly seen as critical to pandemic preparedness and response. Developing research infrastructure and performance may reduce national economies’ reliance on primary commodity and agricultural production, as countries strive to develop knowledge-based economies to help drive macroeconomic growth. Yet efforts to date to understand health sciences research capacity are limited to output metrics of journal citations and publications, failing to reflect the complexity of the health sciences research landscape in many settings.

**Methods:**

We map and assess current capacity for health sciences research across all 54 countries of Africa by collecting a range of available data. This included structural indicators (research institutions and research funding), process indicators (clinical trial infrastructures, intellectual property rights and regulatory capacities) and output indicators (publications and citations).

**Results:**

While there are some countries which perform well across the range of indicators used, for most countries the results are varied—suggesting high relative performance in some indicators, but lower in others. Missing data for key measures of capacity or performance is also a key concern. Taken as a whole, existing data suggest a nuanced view of the current health sciences research landscape on the African continent.

**Conclusion:**

Mapping existing data may enable governments and international organizations to identify where gaps in health sciences research capacity lie, particularly in comparison to other countries in the region. It also highlights gaps where more data are needed. These data can help to inform investment priorities and future system needs.

**Supplementary Information:**

The online version contains supplementary material available at 10.1186/s12961-021-00778-y.

## Introduction

Health sciences research (HSciR) has been defined to include basic, clinical and applied science on human health and well-being, as well as the determinants, prevention, detection, treatment and management of disease [1–3]. To date, the majority of HSciR has taken place in the Global North [4–6]. As of 2018, less than 1% of scientific articles published worldwide each year include at least one author based at an African institution [7].

In the past few years, however, a number of international organizations, including the African Union [8], WHO [9] and the World Bank [10], have called for political and economic investment in HSciR in Africa. Several high-profile reports have further raised awareness of the so-called 10/90 gap: only a 10th of global expenditure on health research is targeted to issues that affect the poorest 90% of the world’s population [5].

There are two key reasons why investments in HSciR in Africa may be particularly important from a developmental perspective. First, the promotion of a strong health science industry, as part of broader efforts to establish a robust research and development (R&D) landscape, can contribute to development goals by reducing national economies’ reliance on primary commodity and agricultural production; this can help governments develop knowledge-based economies, which may be important for macroeconomic growth [11–15]. In a seminal 1990 report, the Commission on Health Research and Development [4] stated that strengthening research capacity in low- and middle-income countries (LMICs) is “one of the most powerful, cost-effective and sustainable means of advancing health and development” (p. 71).

Second, HSciR may contribute to improvements in health, social welfare and poverty reduction through domestic application of the findings of the research itself [16–18]. The 2013 World Health Report stressed that all nations should be producers, users and consumers of HSciR [6]. Africa is home to nearly one sixth of the world’s population and is estimated to account for about a quarter of the global burden of disease [19]. Yet only a small fraction of global health research currently focuses on diseases which exclusively affect LMICs [20, 21]. While there have been developments in the HSciR landscape over the past three decades, many LMICs have been unable to build up sufficient capacity to develop their own evidence base nationally to inform policy directly and/or to improve their population’s health [22–24]. The International Vaccines Task Force of the World Bank has highlighted the importance of building research capacity to lower the risk of emergent epidemics [25].

To date, few academic studies have evaluated HSciR capacity in LMICs. The most widely available indicator of health research capacity is the publication of health-related scientific journal articles. These have been the focus of research in the past, with bibliometric analyses undertaken to map the numbers of African publications related to cardiovascular diseases [26, 27], genomics [28], health economic evaluation [29], health policies and systems [30, 31], human immunodeficiency virus [32], neglected tropical diseases [33] and public health [34]. Four studies have also examined the total number of African publications on any health-related topic (as indexed in major bibliographic databases) [35–38]. Beyond publication outputs, however, researchers have also collected data on investments in health-related R&D [35], clinical trial infrastructures [25, 35], healthcare workforce numbers [39] and the numbers of universities and “centres of excellence” [39, 40] in African countries to estimate HSciR capacity.

Each of these studies can help to understand individual aspects of HSciR capacity in African countries, yet no single piece of information can fully capture the degree of capacity in a country or region. There remains a need for analyses which attempt to collect and analytically combine data on multiple indicators to provide a more comprehensive analysis of HSciR capacity across the continent.

## Background

### The importance of knowledge economies

Science and innovation, if well-utilized, may play a core role in realizing sustainable development [1, 41]. As seen from the experiences of many industrialized nations, scientific research and linked innovations have been core to economic and social advancement over the past two centuries—be it medical innovations such as vaccines and antibiotics, or industrial innovations in manufacturing, communications and computation [42–44].

More recently, questions have been asked as to whether scientific research supports development, or whether it represents a product of development [45, 46]. Both these positions have their justifications. In terms of science resulting in development, it is research and knowledge generation, linked with subsequent innovation and application of that knowledge, that some argue has been critical to overcoming key development challenges in LMICs [45]. Under this position, the need to invest in capacity for mobilizing and using science and innovation can be viewed as an essential component of strategies for promoting sustainable development [47–49]. This argument appears to underpin the inclusion of research within the Sustainable Development Goals. Goal 3.B specifically focusses on health research for LMIC needs, calling for “supporting the development of research and development of vaccines and medicines for health conditions which affect LMICS”; goals 9.5 and 12.A call for increased scientific, technical and research capacity more generally in developing countries [50].

Many calls for the creation of so-called knowledge economies are linked to thinking of research activity as an end goal of development. It has been argued that the conceptualization of an economy of knowledge reproduces a growth and market-oriented rationale for knowledge production, accumulation and diffusion which has particularly influenced the international aid, education and development agenda [51, 52]. For example, the Organisation for Economic Co-operation and Development (OECD) [11] defines knowledge-based economies as those “which are directly based on the production, distribution and use of knowledge and information’’ (p. 7). The World Bank has classified the knowledge economy into four areas: economic and institutional regime, education and skills, information and communication infrastructure, and an innovation system—with the agency going so far as to create a Knowledge Economy Index (KEI) as an indicator of a country’s “preparedness” for a knowledge economy [53].

Asongu and colleagues [54] found the overall trends in African countries’ performance between 1996 and 2010 differed across the World Bank’s KEI dimensions: Tunisia led in education, the Seychelles in information and communication technology, South Africa in innovation and Botswana and Mauritius in institutional regime. Oluwatobi and colleagues [55] have argued that the potential for knowledge production and innovation in Sub-Saharan Africa is mitigated by the level of human capital and quality of institutions. Overall, quality education and strong institutions are held to be imperative for the transformation into a knowledge economy [55–57]. Both educational and economic institutions may create enabling structures for developing knowledge and innovation and for economic growth, but their influence varies according to institutional arrangements, income and development levels in countries [57–59]. In particular, education plays a vital role in strengthening human capital, which directly influences the ability to create, absorb, transform, disseminate and use knowledge and innovation [55, 60–62]. Education and training emphasizing the value of traditional knowledge and culture also strengthens human capital to innovate contextually relevant solutions for local development problems [54, 59, 63].

### The contribution of HSciR

Within the broader remit of science for development, HSciR is vital in its own specific way. HSciR has led to collective human benefit, development of medical treatments, or better understanding of health risks of activities such as tobacco smoking. At a national level, HSciR can also specifically generate evidence that is useful for public service planning and programme implementation. It can provide policy-relevant information, including disease trends, risk factors, outcomes of interventions and patterns of care, as well as health systems and services costs and outcomes [64].

Grant and Buxton developed a framework to estimate the value that HSciR provides to countries [65]. Their analysis has included as benefits reduced expenditure on delivering existing services; service and provision delivery improvements; health service effectiveness improvements; greater overall improvements in health and equity with more consideration of allocation of resources and access to provision; and a healthy, performance-driven workforce.

Finally, Dobrow et al. have shown that HSciR evidence can in turn support development of the process of health policy-making through the identification of new issues worthy of bringing to the policy agenda in a particular context, supporting decision-makers in their analyses of policy content and continued direction and policy impact monitoring and evaluation [66]; Gilson has noted that health policy and systems research more specifically provides insights into how policy decisions are made and the factors affecting successful policy implementation [67]. In many ways, these examples capture the benefits widely seen to follow from a system of evidence-informed policy-making, whereby a more systematic and robust use of research evidence in decision-making is seen to improve planning effectiveness efficiency and policy implementation to serve the broader social good [16, 68].

HSciR input and output by national governments are not uniform, with significant disparities between regions or income levels and also across countries within the same region or at similar levels of income [69, 70]. On the African continent, for example, Tanzania and Lesotho had similar levels of GDP per capita (US$ 2365 and US$ 2494, respectively, in 2013); however, the percentage of GDP invested in research in Tanzania was more than three times as high as in Lesotho (0.28 vs 0.08), while the number of publications per million inhabitants was nearly 50 times as high, at 770 in Tanzania compared to 16 in Lesotho [71].

One of the most critical contextual determinants of HSciR outputs is historical evolution of research systems. For those African nations subject to colonial rule, for instance, modern forms of research were often developed in service to the economic interests of the colonizing power. The focus of research thus centred on key exports such as agriculture-, forestry- and mining-related activity, with little interest in HSciR to benefit local populations [72, 73]. After independence, HSciR remained embryonic, with governments often choosing to invest in economies based on the commercialization of cash products and natural resources rather than in the development of research and technology [74]. Moreover, countries which have experienced conflict, instability and other sociopolitical crises have had to direct resources towards reconstruction and peacekeeping investments, rather than towards scientific research and innovation [75].

For some nations, the catalyst for investment and development of HSciR has mainly been through the emergence of health crises—new diseases such as HIV/AIDS and Ebola, or the rising incidence of tuberculosis and plague [76, 77] (MTN, 2017). Outbreaks have also at times inspired new policies calling for investment in HSciR by global organizations such as WHO and UNESCO [78, 79]. These calls for investment have allowed for a more open dialogue and progress on conceptualizing the importance of health research, even within low-income African states—with several governments now committed to investing in scientific research in connection with a country's economic and sustainable development priorities [80–82]. Despite these shifts, such as the Bamako Initiative, WHO’s efforts to regionalize research efforts and signs of increased attention to domestic HSciR, key drivers of research and research funding in the health sector remain exogenous to African states. Indeed, funding largely reflects global HSciR priorities, with limited options for investigator-initiated research on local health concerns.

### How to measure HSciR?

While there is a strong case that HSciR in LMICs is important at national and global levels—for improving health, preventing epidemic spread, supporting health policy and systems and as an influential factor of national development more broadly—there is no single framework or consensus method to assess HSciR capacity across countries. Indicators for measuring and monitoring HSciR generally include standard output indicators of knowledge production and innovation, such as scientific journal articles per million inhabitants or patents per million inhabitants [53] and input and process indicators of health R&D. Such process indicators can include gross domestic R&D expenditure on health as a percentage of GDP, number of clinical trials per million inhabitants, research grants and full-time equivalent health researcher per million inhabitants [35]. From the perspective of decision-makers in national agencies, these indicators of knowledge production and human resources for research are helpful for benchmarking performance against regional and global comparators and for informing policy and strategy to strengthen R&D [83].

Researchers and international organizations have attempted to compile indicators and measure HSciR capacity in different ways. For example, WHO has created a Global Observatory on Health R&D which aims to “consolidate, monitor and analyse relevant information on health research and development activities” [84]. This uses a logic model perspective to assessing HSciR, tracking a range of indicators to monitor health R&D inputs, processes and outputs as identified and defined by Røttingen and colleagues [35]. While these indicators are useful for monitoring and benchmarking the state of HSciR and development activities, funding and performance at the national level, they do not provide information to assess the overall capacity of national health research systems as a set of “people, institutions and processes” for HSciR [4]. Moreover, there are incomplete or missing data for many of these indicators.

A second approach takes a systems perspective to assessing HSciR capacity, recognizing that R&D funds and personnel represent but two components of a nation’s HSciR capacity. Pang and colleagues [17] defined a national health research system within a conceptual framework considering four key tenets: stewardship, financing, creating and sustaining resources, and producing and using research. This framework has been operationalized under the Research for Health unit at the WHO Regional Office for Africa through the development of a “barometer” that aims to assess the evolution of national health research systems. The team collected data from surveys of individual health research focal points in countries (with rounds in 2003, 2009, 2014 and 2018) [85–89]. Key informants within national ministries of health and other institutions replied to questions about whether HSciR policies, institutions or other resources were currently in place in the country (e.g. national health research policy, national research ethics committee, national health research institute, national budget line for health research) [85].

In applying this approach, Kirigia and colleagues [86] analysed trends between 2003 and 2014 to show that although there have been positive gains across many functions, there are still considerable gaps in many African countries. For example, in sub-Saharan Africa, fewer than 50% of countries have a nationwide official health research policy, or a national health research strategy/policy plan, a HSciR law or regulation, or a much-needed budget line for HSciR within the ministry of health. Approximately half of states analysed have a national health research institute/council, a research programme at the governmental level, or an equivalent health research management forum. Public financing for HSciR is also typically measured to be very low, with minimal progress towards the goal of 2% of the national health expenditure allocated to HSciR. Instead, most funds for HSciR come from external sources such as international organizations, nongovernmental organizations (NGOs) or multilateral/bilateral partners. According to Kirigia et al., the weakest elements of African health research systems are human resources for HSciR, government spending on HSciR, publications in peer-reviewed journals, and research institutions to conduct HSciR [87].

Overall, there have been a variety of attempts to identify key elements of HSciR activity, performance and capacity in Africa. Some have assessed R&D potential, measured funding inputs or identified gaps in national HSciR systems. These efforts shed light on where strengths and weaknesses lie, but currently do not provide a comprehensive review and synthesis of data on which to comparatively evaluate HSciR knowledge and innovation, HSciR and development activities and HSciR systems at the national level across Africa.

The aim of this paper is to build on earlier work by collecting and aggregating data on a range of variables to consider HSciR activity, performance and capacity in all African countries. We develop a framework for evaluating a country’s capacity for HSciR based on publicly available global data sources. This framework incorporates and expands on indicators from previous studies. Using this framework, we present data on HSciR capacity in each of the 54 UN-recognized African states to map current capacity across the region for HSciR—providing one of the first analyses to systematically outline the contribution of African countries to HSciR across such a wide range of indicators.

## Methods

### Data collection

We reviewed data for each of the 54 UN-recognized states in Africa. This excluded any foreign departments (e.g. Mayotte), regions (e.g. Réunion) or territories (e.g. Saint Helena) located in Africa, as well as the disputed territory of Western Sahara. We collected population and gross domestic product (GDP) data from the World Bank [90] for each of these states to be able to benchmark our findings against broader development metrics.

We sought to identify a range of indicators which could help measure the HSciR capacity in each country. We used the indicators selected by the WHO Global Observatory on Health R&D database as a starting point, which comprised GERD as a proportion of GDP, health researchers per million inhabitants, number of institutions and official development assistance for the medical research and basic health sectors as a proportion of gross national income [91]. We then supplemented this with others measures of HSciR capacity which we identified through discussions between authors and members of a project oversight committee,^1^ including bibliographic data, data on clinical trial infrastructures, regulatory environment, intellectual property rights and research funding. All data were acquired between June and September 2018.

To classify and conceptualize the various indicators available, we followed the Donabedian [92] model of healthcare quality measurement to categorize our indicators into one of three types: structural, process and output measures related to HSciR. Structural measures capture inputs into the system and thus comprised metrics such as workforce numbers, budget allocation to R&D and numbers of organizations, regulations and guidelines on human subject protections. Process measures are indicators of ongoing HSciR activities, including numbers of clinical trials registered and patent applications. Finally, output measures capture the outputs of research activities including numbers of peer-reviewed publications and citations for these publications.

### Structural indicators

#### R&D expenditures and personnel

Data on R&D expenditure and personnel were obtained from the United Nations Educational, Scientific and Cultural Organization (UNESCO) (2016, or the most recent available year) [91]. We collected data on the number of full-time-equivalent staff in the following categories: (i) R&D personnel (per million inhabitants), (ii) researchers (per million inhabitants) and (iii) researchers with doctoral or equivalent degrees (as a proportion of total number of researchers). From the same database, we also collected data on GERD in current purchasing power parity (PPP) dollars (in thousands); these figures were also shown as a proportion of GDP and per capita. Whenever possible, we collected expenditure and personnel data specific to medical and health sciences.

#### Research institutions

We collected data on the number of universities in each country, using a list based on information from the International Association of Universities [93]. We recognize that there may be limitations affecting the quality of data from this source; thus, we also identified the number of African universities listed on the most recent global university rankings of three influential publishers: Quacquarelli Symonds Limited (*QS World University Rankings*) [94], *Times Higher Education* (*THE World University Rankings*) [95] and Shanghai Ranking Consultancy (*Academic Ranking of World Universities*) [96]. Whilst this may not be comprehensive, it allows an indication of the number of institutions across the continent.

We further collected data on the number of institutional review boards [97] and WHO Collaborating Centres [98] in each country and noted whether or not there exists a national ethics committee [99] and national public health institute [100].

### Research funding

We collected data on international funding awarded to researchers in each country (2008–2017) from the 10 largest public and philanthropic funders of health research globally (listed in order of size) [101]: (1) United States National Institutes of Health, (2) European Commission, (3) United Kingdom Medical Research Council, (4) French National Institute of Health and Medical Research, (5) United States Department of Defense (including the Congressionally Directed Medical Research Programs), (6) Wellcome Trust, (7) Canadian Institutes of Health Research, (8) Australian National Health and Medical Research Council, (9) Howard Hughes Medical Institute and (10) German Research Foundation.

The data were collected from each funder’s website. As we are seeking to understand current capacity, we only counted funding allocated to researchers based at institutions in African countries. We excluded funding for research projects in which the principal investigators were based at non-African institutions, even if these projects included collaborators, field sites or locations of research in Africa.

Foreign currencies were converted to dollars based on the yearly average exchange rates published by the World Bank [90]. All amounts were reported in 2018 US dollars based on the United States consumer price index adjustments to account for inflation.

#### Process indicators (clinical trial infrastructures, intellectual property rights and regulatory capacities)

Data on the numbers of clinical trials and records, as of 4 August 2018, were extracted from the WHO International Clinical Trials Registry Platform (ICTRP) [102] and United States National Institutes of Health database (ClinicalTrials.gov) [103]. ClinicalTrials.gov indexes trials of new investigational drugs, whereas the ICTRP indexes data from several sources, including the European Union Clinical Trials Register, ClinicalTrials.gov, International Standard Randomised Controlled Trial Number register and Pan African Clinical Trial Registry. A full list of data providers can be found on the ICTRP website [102]. The ICTRP registry accepts all types of clinical research studies, including trials of public health interventions.

We also collected information on the number of organizations, regulations and guidelines on human subjects protection in each country. These data, which are collected annually by the United States Department of Health and Human Services [104], reflect protections in each of the following categories: “general (i.e. applicable to most or all types of human subjects research)”, “drugs and devices”, “clinical trial registries”, “research injury”, “social-behavioural research”, “privacy/data protection”, “human biological materials”, “genetic” and “embryos, stem cells and cloning”. We used the 2018 edition of the compilation of protections [104].

Finally, we collected data from the World Intellectual Property Organization on the numbers of patents issued to residents in each country (2016, or most recent available year) [105].

### Output indicators (publications and citations)

To systematically collect publication data, we searched Scopus, the largest global peer-reviewed literature abstract and citation database [106]. Scopus was chosen as it includes a larger volume of non-English-language journals than many other major bibliographic databases (e.g. Web of Science or PubMed/Medline) [106]. We searched for any articles published in the following Scopus subject areas: health sciences (medicine, nursing, veterinary, dentistry, health professions) and life sciences (agricultural and biological sciences, biochemistry, genetics and molecular biology, immunology and microbiology, neuroscience and pharmacology, toxicology and pharmaceutics). We included the following types of publications: articles, in press, books, chapters and conference papers.

We searched for articles published with at least one author based at an institution in each of the 54 countries, using the “Affiliation country” field in Scopus. We searched the names of each country in English, French and Portuguese, as well as variant spellings of country names. We restricted the searches to publications published in the 10-year period from 2008 to 2017. The search strategy, including the country names, can be found in Additional file 1.

For each country, we extracted data on the number of publications with at least one author based in the country, as well as the number of publications first authored by a local researcher. We also collected citation data for all articles. For publications published in the 5-year period from 2013 to 2017, we collected data on the proportion of publications with international, institutional and national collaborators; these data were unavailable for articles published before 2013. These data were obtained in SciVal, a research information tool developed by Elsevier to synthesize bibliometric data from Scopus.

## Results

Data for each individual indicator are presented as a series of tables in Additional file 2. We describe findings for each indicator in Additional file 3, before providing a summary table in this section (see Table 1).

**Table 1:**
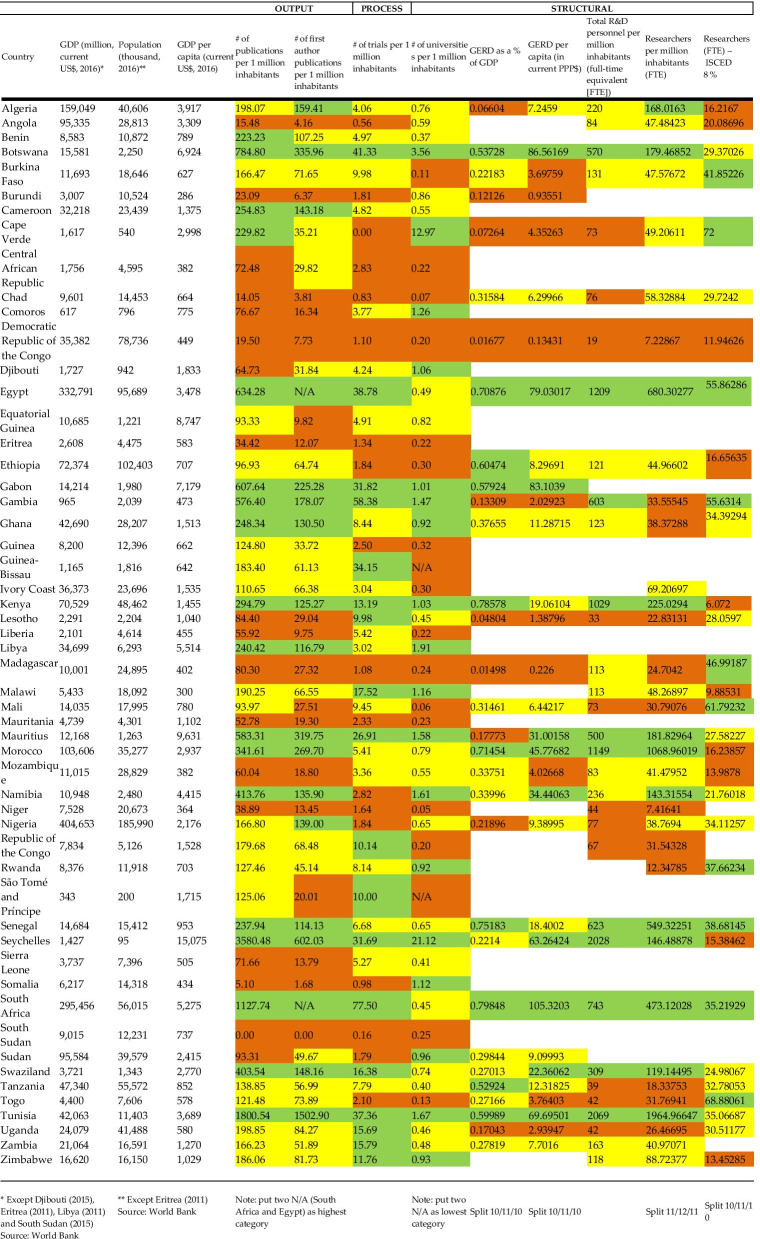
Indicators framework for all 54 sovereign African States

*Except Djibouti (2015), Eritrea (2011), Libya (2011) and South Sudan (2015) Source: World Bank

**Except Eritrea (2011) Source: World Bank

Put two N/A (South Africa and Egypt) as highest category

Put two N/A as lowest category

Split 10/11/10

Split 10/11/10

Split 11/12/11

Split 10/11/10

Table 1 below presents data for selected indicators. We have shaded each cell to reflect whether the data in that cell fall in the highest, middle or lowest tercile of the range, with green for the top tercile, yellow the middle and orange the bottom. The table shows that while there were some high achievers across the board, the results were varied for most states—suggesting high relative performance in some indicators, but lower in others (or missing data). For example, Libya was a relatively high achiever for publications, first author publications and number of research institutions, but lagged behind this success with the number of clinical trials conducted within the country. Conversely, Burundi had low numbers of publications, first author publications, number of clinical trials and GERD as a percentage of GDP, but performed relatively well in number of research institutions. While many of the higher-income countries unsurprisingly perhaps do well on numerous indicators, some lower-income countries also appear to perform well, such as Senegal or Kenya.

Additional file 2 presents a further set of 10 figures to illustrate associations between various metrics and GDP (gross and per capita). In general, there was a strong positive association between GDP and the various indicators collected. However, there could be significant spread around the linear trend lines plotted, indicating some countries performing particularly well or poorly relative to income level.

### Limitations

This study has limitations. First, there is no single indicator for accurately ascertaining HSciR. Accordingly, we used a variety of available metrics that serve as proxy indicators. Thus, there is a risk that these proxies do not capture the full landscape we sought to map. For example, we have not accounted for broader financing and infrastructure which contributes to HSciR—such as buildings, routine access to electricity, and primary and secondary education attainment. Each of these may contribute to a country’s capacity for HSciR, but they may be part of broader development measures. Such indicators may not clearly demonstrate the impact of HSciR and can be difficult to disaggregate. However, there is support for the indicators we selected for our framework from the literature discussing measurement of health R&D globally and in Africa [35, 107]. Nevertheless, we acknowledge that there are multiple challenges and issues with measuring HSciR performance using universal indicators in the contexts of African national health research systems [108].

Second, there are some important limitations of the sources of data used. Given the lack of consistent data on HSciR collected and reported at national levels, or indeed at the regional level, we restricted our searches to global-level data sets to try to ensure some degree of comparability. This approach fails to take into account the reality of what might be occurring within countries which is not reported formally or is not published at the national, regional or global level. Moreover, at the global level there was a lack of data for several indicators, or these data were outdated. The most comprehensive data sources were for publications and clinical trials, but many other indicators were missing results for numerous countries. In some data categories, there were issues of reliability and comparability between sources. Furthermore, while we aimed to collect data from 2007 to 2017, some data points had to come from before this time frame when more recent data were not available. This included data on patent applications and human resources. Ultimately, we decided that including data outside the period was better to get a fuller picture of the HSciR landscape.

Third, for the output indicators, it is important to note that research outputs are not always published in peer-reviewed journals; therefore, limiting the analysis to bibliometrics from SciVal could have led us to outputs in other sources and formats. Our approach does not include research published outside of peer-reviewed journals, including government or nongovernmental literature policy reports, open data sets, software or other grey literature. Scopus also does not index all journals published in African states. Similarly, we recognize that for the structural indicators, we only included universities, which may provide an incomplete picture of key research structures and institutions within a country. While the presence of universities was felt to be a reasonably comparable metric to serve as a proxy for infrastructure capacity, it is known that there are very important contributors to the HSciR landscape in Africa that are not affiliated to a specific university per se—for example, Tanzania’s Ifakara Health Institute, the Kenya Medical Research Institute and the African Population and Health Research Centre (APHRC). These entities undertake a large amount of work at national or regional levels. Indeed, some centres of excellence in Africa may be undertaking a very large share of HSciR in a given country, and not be affiliated with a university. While the activities of such organizations might be captured in publication metrics or clinical trials data, future efforts to evaluate HSciR capacity may need to consider ways to identify, count or compare the importance of centres of excellence as core hubs of institutional capacity [40].

Fourth, we were unable to find a consistent data source across the African continent to measure government budget allocation to HSciR. Instead, we used GERD as a proxy, which (see Table 1) captures investments in R&D (although not disaggregated by health sciences). Furthermore, data on GERD aggregate total expenditure on R&D from the government as well as university, private enterprise and not-for-profit sectors; yet this breakdown is rarely available for many African countries. The data available through internationally recognized and consistent sources on GERD for medical and health sciences are sparse (see Additional file 2: Table S4). The WHO African Barometer survey collects data on health research budgets, which is self-reported by health research focal points at ministries of health. Data from 2018 reported that 24 countries had a dedicated budget line for health research and that 37 countries regularly tracked health research spending from all sources [89]. Whilst these data show a limited scope of HSciR funding, taking only the ministry of health budget and not other sources, it could contribute to better understanding of HSciR funding in countries. These data have not been made publicly available by intergovernmental sources, and we found no centralized data on national research funds on the continent in any comparable way, although in-depth qualitative case studies in a sample of nine African countries found these in five instances [109]. Future work evaluating HSciR could investigate which countries of the region have such funds, as there does not appear to be any data source indicating it at present despite a nascent literature on the topic [110–113]. Similarly, when measuring the number of universities in each state, we were not able to ascertain whether these universities undertake research or solely offer degrees or training in health sciences.

Fifth, these metrics are all aggregated at the national level, and thus this crude analysis fails to reveal any subnational interaction. A more in-depth analysis could reveal particular “hubs” of excellence, as well as institutional capacity or individual capacities which form key components of the national landscape.

## Discussion

Taken as a whole, the existing data we were able to analyse offer a nuanced view of current HSciR on the African continent. We hope that such mapping facilitates governments and international organizations in identifying gaps in HSciR capacity, particularly in comparison to other countries in the region, if important. Our findings importantly also highlight gaps where more data are very much needed. These data can help to inform investment priorities and future system needs.

Our findings have raised several issues for consideration:

There are some unsurprising high performers across the variety of indicators, such as South Africa, Egypt and Tunisia, which score highly across most metrics. However, it is worth noting that it is not simply the level of development (GDP) or international or national financing for HSciR (GERD and international research funding) that leads to success in HSciR. Nations which have had major donor investment in HSciR (per capita), including Uganda and Gambia, have not necessarily emerged as top performers across the range of proxy indicators used. Whilst the current level of economic development does not appear to play a significant role in a country’s HSciR capacity per se, our analysis shows a clear correlation between GDP and a range of individual metrics (Additional file 2), although this is not evidence of causality).

There are several possible explanations for these results. One explanation might be that reliance on donor funding has limited the sustainability of the health research sector when these collaborations end [114, 115], or that donor investment focused on projects which lacked significant improvements in broader infrastructures within the national system. Alternatively, international arrangements may result in research agendas set by the Global North, which could imply that they either reflect the needs of the funding location [116, 117], a focus on spotlight issues [118] or so-called parachute research [119, 120] and bypass local research institutions and expertise [121], any of which may be limited in improving health research outcomes or capacity in the host location. The importance of local research development, however, has been highlighted as vital to building a knowledge economy and addressing domestic health concerns, as in-country researchers have the best understanding of the national agenda and cultural context which increased the likelihood of evidence uptake by policy-makers [22, 122]. Yet, it is clear that several African governments have not met the commitment to ensure that 1% GDP is dedicated to research, and many have struggled to make even minimal investments in HSciR from public finances, being more reliant on international donors or private entities.

Another explanation is that using these indicators to measure performance does not capture the nuance of what is occurring within each system, particularly within each nation and the progress that research systems are making more holistically. For example, these metrics are not able to infer political commitment to HSciR, the relative importance of the HSciR landscape globally, how national systems have developed, where the success stories are and where barriers remain to solidifying knowledge economies. They are also unable to infer the historical contexts which led to the development of these systems, whether rooted in colonial science or postcolonial investments, each of which will lead to different-looking HSciR environments.

Moreover, there is a paucity of national data provided by governments across the African continent in the public domain. This was a notable gap and challenge for assessing the landscape of HSciR across the region. There may be few incentives in place to do so, but it would be important for governments to make more national-level data available for future studies. This would allow future research to provide a realistic picture of HSciR within each nation and thus be able to make meaningful assessments of country capacity or areas for future investment. Our hope is that with this mapping exercise, whilst limited to global data sources, governments will be able to identify where their gaps in HSciR lie, or their perceived relative performance compared to regional counterparts. This may offer meaningful analysis for investment priorities and future health research system needs.

## Conclusion

There has been a growing awareness of the importance of building health research systems in lower-income settings over time, whether it is to provide useful evidence to inform policy and health systems performance, to develop so-called knowledge economies that support economic growth, or to ensure that there are research systems in place that can assist countries in responding to emergent and novel epidemic threats such as COVID-19. A range of stakeholders, including national governments, international donor agencies and global health policy-making bodies, may all be increasingly interested in ways to evaluate HSciR to guide future developments. We have sought to assess current capacity for HSciR across Africa based on a subset of proxy indicators for which we had more complete data considering structural, process and output indicators. In doing so, we contribute to quantifying current strengths and lack of capacity in the HSciR landscape. Importantly, we did not find particular differential trends between these indicators. Some countries performed well across all three types of indicators, with variation amongst those performing less well as to where strengths lay— there were some locations which had stronger output indicators, but this did not necessarily correlate to strong process and structural drivers, and vice versa.

## Supplementary Information

**Additional file 1.** Search strategy and terms.

**Additional file 2: Table S1.** Bibliometric data. **Table S2.** Clinical trial infrastructures and intellectual property rights. **Table S3.** R&D personnel. **Table S4.** R&D expenditure. **Table S5.** Regulatory capacities. **Table S6.** Funding. **Table S7.** Regression summary for gross domestic product and number of publications. **Figure S1.** The relationship between gross domestic product and publications. **Figure S2.** The relationship between gross domestic product per capita and publications per capita. **Table S8.** Regression summary for gross domestic product per capita and the number of publications per capita. **Figure S3.** The relationship between gross domestic product and patent applications. **Table S9.** Regression summary for gross domestic product and patent applications. **Figure S4.** The relationship between gross domestic product per capita and patent applications per capita. **Table S10.** Regression summary for gross domestic product per capita and patent applications per capita. **Figure S5.** The relationship between gross domestic product and GERD. **Table S11.** Regression summary for gross domestic product and GERD. **Figure S6.** The relationship between gross domestic product per capita and GERD per capita. **Table S12.** Regression summary for gross domestic product per capita and GERD per capita. **Figure S7.** The relationship between gross domestic product and universities. **Table S13.** Regression summary for gross domestic product and universities. **Figure S8.** The relationship between gross domestic product per capita and universities per capita. **Table S14.** Regression summary for gross domestic product per capita and universities per capita. **Figure S9.** The relationship between gross domestic product and clinical trials. **Table S15.** Regression summary for gross domestic product and clinical trials. **Figure S10.** The relationship between gross domestic product per capita and clinical trials per capita. **Table S16.** Regression summary for gross domestic product per capita and clinical trials per capita.

**Additional file 3.** Description of Additional file 2 Tables S1–S6.

## Data Availability

All data generated or analysed during this study are included in this published article and its additional information files.

## Abbreviations

APHRC: African Population and Health Research Centre
GDP: Gross domestic product
GERD: Gross domestic expenditure on R&D
HSciR: Health sciences research
ICTRP: International Clinical Trials Registry Platform
KEI: Knowledge Economy Index
LMICs: Low- and middle-income countries
PPP: Purchasing power parity
R&D: Research and development
UNESCO: United Nations Educational, Scientific and Cultural Organization

## Footnotes

1 This project oversight group comprised funders, researchers and policy officers based in Kenya, Ghana, South Africa, the United States and the United Kingdom, and was convened by Wellcome Trust.
