# Supplementary material for: Measuring health science research and development in Africa: mapping the available data
Source: Health Res Policy Syst. 2021 Dec 11;19:142. doi: 10.1186/s12961-021-00778-y (PMC8665309; doi:10.1186/s12961-021-00778-y)
Supplement: Supplementary file 1 — Additional file 1. Search strategy and terms. [file 12961_2021_778_MOESM1_ESM.docx]

#### **Additional file 1. Search strategy and terms**

1. SUBJAREA(medi OR nurs OR vete OR dent OR heal OR mult OR agri OR bioc OR immu OR neur OR phar)

2. DOCTYPE(ar OR ip OR bk OR ch OR cp)

3. PUBYEAR AFT 2007

4. PUBYEAR BEF 2018

5. AFFILCOUNTRY([insert the country lists below individually])

6. 1 AND 2 AND 3 AND 4 AND 5

For the Republic of Congo and Democratic Republic of Congo, we included an additional filter based on city, as shown below. This was to differentiate between the two Congos. We used the five most populous cities in each country, based on recent estimates.

For South Sudan, we also included an additional filter based on affiliation city. This was because many of the South Sudanese publications were incorrectly classified as Sudanese. We also only included publications published from 2011 onwards (year of independence).

In the searches, we enclosed the names of countries consisting of more than one word with curly brackets, rather than quotation marks, to improve the accuracy of the searches. The names are shown below with quotation marks for ease of reading.

* These entries show the exact Scopus code for line 5 in the search. In the South Sudan search, the PUBYEAR was changed to after 2010 in line 3 of the search, since the country gained independence from Sudan in 2011.

| **Number** | **Country terms** |
| --- | --- |
| 1 | (Algeria OR Algérie OR Argélia) |
| 2 | (Angola) |
| 3 | ((Benin OR Bénin OR Benim) AND NOT (Nigeria OR Nigéria)) |
| 4 | (Botswana) |
| 5 | (“Burkina Faso”) |
| 6 | (Burundi) |
| 7 | (Cameroon OR Cameroun OR Camarões) |
| 8 | (“Cape Verde” OR “Cap-Vert” OR “Cabo Verde”) |
| 9 | (“Central African Republic” OR “République centrafricaine” OR “República Centro-Africana”) |
| 10 | (Chad OR Tchad OR Chade) |
| 11 | (Comoros OR Comores) |
| 12* | (AFFILCOUNTRY((Brazzaville OR “Congo Brazzaville” or “Congo-Brazzaville” OR “República do Congo” OR “République du Congo” OR “Congo Republic”) AND NOT (Zaire OR “Democratic Republic of the Congo” OR “DR Congo” OR “République démocratique du Congo” OR “República Democrática do Congo” OR “Congo-Kinshasa” OR “Congo Kinshasa” OR “DRC” OR “Democratic Republic Congo” OR “Democratic Republic of Congo”)) OR (AFFILCOUNTRY(“Congo” OR “The Congo”) AND AFFILCITY(“Brazzaville” OR “Pointe-Noire” OR “Dolisie” OR “Nkayi” OR “Kindamba”))) |
| 13 | (“Côte d'Ivoire” OR “Ivory Coast” OR “Costa do Marfim”) |
| 14* | (AFFILCOUNTRY((Zaire OR “Democratic Republic of the Congo” OR “DR Congo” OR “République démocratique du Congo” OR “República Democrática do Congo” OR “Congo-Kinshasa” OR “Congo Kinshasa” OR “DRC” OR “Democratic Republic Congo” OR “Democratic Republic of Congo”) AND NOT (Brazzaville OR “Congo Brazzaville” or “Congo-Brazzaville” OR “República do Congo” OR “République du Congo” OR “Congo Republic”)) OR (AFFILCOUNTRY(“Congo” OR “The Congo”) AND AFFILCITY(“Kinshasa” or “Lubumbashi” OR “Mbuji-Mayi” OR “Bukavu” OR “Kananga”))) |
| 15 | (Djibouti) |
| 16 | (Egypt OR Égypte OR Egito) |
| 17 | (Eritrea OR Érythrée OR Eritreia) |
| 18 | (Ethiopia OR Éthiopie or Etiópia) |
| 19 | (“Equatorial Guinea” OR “Guinée équatoriale” OR “Guiné Equatorial”) |
| 20 | (Gabon or Gabão) |
| 21 | (Gambia OR Gambie OR Gâmbia) |
| 22 | (Ghana OR Gana) |
| 23 | ((Guinea OR Guinée OR Guiné) AND NOT (“Guinea-Bissau” OR “Guiné-Bissau” OR “Guinea Bissau” OR “Guinée-Bissau” OR “Equatorial Guinea” OR “Guinée équatoriale” OR “Guiné Equatorial”)) |
| 24 | (“Guinea-Bissau” OR “Guinée-Bissau” OR “Guiné-Bissau”) |
| 25 | (Kenya OR Quénia) |
| 26 | (Lesotho OR Lesoto) |
| 27 | (Liberia OR Libéria) |
| 28 | (Libya OR Libye OR Líbia OR “Libyan Arab Jamahiriya”) |
| 29 | (Madagascar OR Madagáscar) |
| 30 | (Malawi) |
| 31 | (Mali) |
| 32 | (Mauritania OR Mauritanie OR Mauritânia) |
| 33 | (Mauritius OR Maurice OR Maurícia) |
| 34 | (Morocco OR Maroc) |
| 35 | (Mozambique OR Moçambique) |
| 36 | (Namibia OR Namibie OR Namíbia) |
| 37 | ((Niger OR Níger) AND NOT (Nigeria OR Nigéria)) |
| 38 | (Nigeria OR Nigéria) |
| 39 | (Rwanda OR Ruanda) |
| 40 | (“Sao Tome and Principe” OR “São Tomé and Príncipe” OR “São Tomé-et-Principe” OR “São Tomé e Príncipe”) |
| 41 | (Senegal OR Sénégal) |
| 42 | (Seychelles) |
| 43 | (“Sierra Leone” OR “Serra Leoa”) |
| 44 | (Somalia OR Somalie OR “Somália”) |
| 45 | (“South Africa” OR “Afrique du Sud” OR “África do Sul”) |
| 46* | AFFILCOUNTRY(“South Sudan” OR “Soudan du Sud” OR “Sudão do Sul”) OR (AFFILCITY(Juba) |
| 47* | (AFFILCOUNTRY(Sudan OR Soudan OR Sudão) AND NOT AFFILCOUNTRY(“South Sudan” OR “Soudan du Sud” OR “Sudão do Sul”)) AND NOT (AFFILCITY(Juba) AND PUBYEAR > 2010) |
| 48 | (Swaziland OR Swasiland OR Suazilândia) |
| 49 | (Tanzania OR Tanzanie OR Tanzânia) |
| 50 | (Togo) |
| 51 | (Tunisia OR Tunisie OR Tunísia) |
| 52 | (Uganda OR Ouganda) |
| 53 | (Zambia OR Zambie OR Zâmbia) |
| 54 | (Zimbabwe) |
