# Supplementary material for: Measuring health science research and development in Africa: mapping the available data
Source: Health Res Policy Syst. 2021 Dec 11;19:142. doi: 10.1186/s12961-021-00778-y (PMC8665309; doi:10.1186/s12961-021-00778-y)
Supplement: Supplementary file 2 — Additional file 2: Table S1. Bibliometric data. Table S2. Clinical trial infrastructures and intellectual property rights. Table S3. R&D personnel. Table S4. R&D expenditure. Table S5. Regulatory capacities. Table S6. Funding. Table S7. Regression summary for gross domestic product and number of publications. Figure S1. The relationship between gross domestic product and publications. Figure S2. The relationship between gross domestic product per capita and publications per capita. Table S8. Regression summary for gross domestic product per capita and the number of publications per capita. Figure S3. The relationship between gross domestic product and patent applications. Table S9. Regression summary for gross domestic product and patent applications. Figure S4. The relationship between gross domestic product per capita and patent applications per capita. Table S10. Regression summary for gross domestic product per capita and patent applications per capita. Figure S5. The relationship between gross domestic product and GERD. Table S11. Regression summary for gross domestic product and GERD. Figure S6. The relationship between gross domestic product per capita and GERD per capita. Table S12. Regression summary for gross domestic product per capita and GERD per capita. Figure S7. The relationship between gross domestic product and universities. Table S13. Regression summary for gross domestic product and universities. Figure S8. The relationship between gross domestic product per capita and universities per capita. Table S14. Regression summary for gross domestic product per capita and universities per capita. Figure S9. The relationship between gross domestic product and clinical trials. Table S15. Regression summary for gross domestic product and clinical trials. Figure S10. The relationship between gross domestic product per capita and clinical trials per capita. Table S16. Regression summary for gross domestic product per capita and clinical trials per capita. [file 12961_2021_778_MOESM2_ESM.docx]

# Additional file 2 – Phase 1 results tables and figures

# Table S1: Bibliometric data

Country

|  | (million) | (thousand) |  |  | pubs. | cit. | pubs. | pubs. | Coll. | Coll. | Coll. | author |
| --- | --- | --- | --- | --- | --- | --- | --- | --- | --- | --- | --- | --- |
| Algeria | 159,049 | 40,606 | 8,043 | 57,188 | 6,473 | 28,931 | 437 | 1,138 | 52.7 | 10.1 | 34.4 | 2.7 |
| Angola | 95,335 | 28,813 | 446 | 4,827 | 120 | 489 | 21 | 85 | 92.5 | 0.3 | 7.2 | 0.0 |
| Benin | 8,583 | 10,872 | 2,427 | 22,042 | 1,166 | 7,435 | 161 | 290 | 80.7 | 1.0 | 17.8 | 0.6 |
| Botswana | 15,581 | 2,250 | 1,766 | 28,298 | 756 | 4,583 | 113 | 245 | 79.7 | 3.5 | 12.9 | 3.8 |
| Burkina Faso | 11,693 | 18,646 | 3,104 | 36,231 | 1,336 | 8,663 | 216 | 388 | 84.5 | 0.8 | 14.2 | 0.4 |
| Burundi | 3,007 | 10,524 | 243 | 2,250 | 67 | 311 | 6 | 40 | 87.4 | 0.0 | 11.4 | 1.2 |
| Cameroon | 32,218 | 23,439 | 5,973 | 69,384 | 3,356 | 21,499 | 376 | 814 | 73.7 | 7.5 | 17.0 | 1.8 |
| Cape Verde | 1,617 | 540 | 124 | 1,052 | 19 | 52 | 6 | 20 | 94.4 | 1.1 | 3.3 | 1.1 |
| Central African Republic | 1,756 | 4,595 | 333 | 4,490 | 137 | 797 | 19 | 40 | 77.7 | 1.0 | 15.8 | 5.4 |
| Chad | 9,601 | 14,453 | 203 | 2,105 | 55 | 357 | 15 | 41 | 94.8 | 0.0 | 5.2 | 0.0 |
| Comoros | 617 | 796 | 61 | 419 | 13 | 33 | 2 | 7 | 77.8 | 2.8 | 19.4 | 0.0 |
| Democratic Republic of the Congo | 35,382 | 78,736 | 1,535 | 26,099 | 609 | 3,330 | 64 | 261 | 86.2 | 0.5 | 12.2 | 1.2 |
| Djibouti | 1,727 | 942 | 61 | 419 | 30 | 166 | 2 | 7 | 77.8 | 2.8 | 19.4 | 0.0 |
| Egypt | 332,791 | 95,689 | 60,693 | - | - | - | 2,481 | 8,206 | 46.4 | 14.1 | 32.7 | 6.9 |
| Equatorial Guinea | 10,685 | 1,221 | 114 | 1,714 | 12 | 43 | 6 | 12 | 100.0 | 0.0 | 0.0 | 0.0 |
| Eritrea | 2,608 | 4,475 | 154 | 1,252 | 54 | 176 | 15 | 18 | 83.3 | 0.0 | 14.6 | 2.1 |
| Ethiopia | 72,374 | 102,403 | 9,926 | 95,131 | 6,630 | 43,786 | 398 | 1,768 | 57.1 | 6.5 | 32.2 | 4.2 |
| Gabon | 14,214 | 1,980 | 1,203 | 20,122 | 446 | 6,199 | 84 | 141 | 89.5 | 0.0 | 9.3 | 1.1 |
| Gambia | 965 | 2,039 | 1,175 | 32,033 | 363 | 6,243 | 92 | 156 | 95.7 | 0.3 | 2.0 | 2.0 |
| Ghana | 42,690 | 28,207 | 7,005 | 96,341 | 3,681 | 19,958 | 307 | 1,118 | 68.7 | 5.1 | 22.6 | 3.6 |
| Guinea | 8,200 | 12,396 | 1,547 | 28,939 | 418 | 3,416 | 126 | 231 | 91.2 | 0.2 | 6.4 | 2.2 |
| Guinea-Bissau | 1,165 | 1,816 | 333 | 5,016 | 111 | 1,963 | 18 | 48 | 99.1 | 0.5 | 0.0 | 0.5 |
| Ivory Coast | 36,373 | 23,696 | 2,622 | 31,674 | 1,573 | 5,637 | 251 | 284 | 67.8 | 1.1 | 30.4 | 0.7 |

GDP

Pop.

Pubs. Cit.

F-a

F-a

2008

2017

Int.

Nat.

Inst.

Single

**Table S1:** Bibliometric data

Country

|  | (million) | (thousand) |  |  | pubs. | cit. | pubs. | pubs. | Coll. | Coll. | Coll. | author |
| --- | --- | --- | --- | --- | --- | --- | --- | --- | --- | --- | --- | --- |
| Kenya | 70,529 | 48,462 | 14,286 | 243,026 | 6,071 | 61,021 | 943 | 1,909 | 83.2 | 5.6 | 9.0 | 2.2 |
| Lesotho | 2,291 | 2,204 | 186 | 2,581 | 64 | 478 | 14 | 22 | 88.6 | 0.0 | 9.5 | 1.9 |
| Liberia | 2,101 | 4,614 | 258 | 3,733 | 45 | 296 | 7 | 57 | 92.8 | 1.4 | 3.4 | 2.4 |
| Libya | 34,699 | 6,293 | 1,513 | 14,307 | 735 | 3,379 | 77 | 151 | 81.3 | 0.2 | 14.9 | 3.5 |
| Madagascar | 10,001 | 24,895 | 1,999 | 23,688 | 680 | 4,113 | 157 | 264 | 86.2 | 0.3 | 12.5 | 0.9 |
| Malawi | 5,433 | 18,092 | 3,442 | 56,398 | 1,204 | 13,344 | 228 | 540 | 90.5 | 1.1 | 6.0 | 2.3 |
| Mali | 14,035 | 17,995 | 1,691 | 26,428 | 495 | 4,014 | 114 | 244 | 89.9 | 0.3 | 9.4 | 0.4 |
| Mauritania | 4,739 | 4,301 | 227 | 1,719 | 83 | 400 | 13 | 32 | 90.9 | 0.0 | 8.4 | 0.7 |
| Mauritius | 12,168 | 1,263 | 737 | 8,004 | 404 | 2,294 | 37 | 104 | 65.3 | 0.2 | 30.4 | 4.0 |
| Morocco | 103,606 | 35,277 | 12,051 | 80,662 | 9,514 | 32,877 | 744 | 1,537 | 36.8 | 14.2 | 47.3 | 1.8 |
| Mozambique | 11,015 | 28,829 | 1,731 | 39,885 | 542 | 5,014 | 81 | 290 | 93.4 | 1.2 | 3.8 | 1.6 |
| Namibia | 10,948 | 2,480 | 1,026 | 10,156 | 337 | 1,386 | 44 | 154 | 83.9 | 0.4 | 8.3 | 7.4 |
| Niger | 7,528 | 20,673 | 804 | 9,075 | 278 | 1,808 | 56 | 99 | 84.7 | 1.3 | 12.4 | 1.7 |
| Nigeria | 404,653 | 185,990 | 31,023 | 205,135 | 25,853 | 109,386 | 2,443 | 3,369 | 36.3 | 14.1 | 45.1 | 4.5 |
| Republic of the Congo | 7,834 | 5,126 | 921 | 11,026 | 351 | 1,496 | 50 | 102 | 80.9 | 0.3 | 17.1 | 1.6 |
| Rwanda | 8,376 | 11,918 | 1,519 | 23,953 | 538 | 4,022 | 50 | 245 | 90.1 | 0.5 | 7.1 | 2.4 |
| Sao Tome and Principe | 343 | 200 | 25 | 335 | 4 | 56 | 0 | 7 | 100.0 | 0.0 | 0.0 | 0.0 |
| Senegal | 14,684 | 15,412 | 3,667 | 37,424 | 1,759 | 10,138 | 234 | 473 | 73.1 | 4.5 | 21.1 | 1.3 |
| Seychelles | 1,427 | 95 | 339 | 5,631 | 57 | 567 | 31 | 44 | 83.0 | 0.5 | 4.5 | 2.0 |
| Sierra Leone | 3,737 | 7,396 | 530 | 7,489 | 102 | 975 | 15 | 114 | 94.5 | 0.2 | 4.2 | 1.0 |
| Somalia | 6,217 | 14,318 | 73 | 685 | 24 | 74 | 3 | 21 | 96.2 | 0.0 | 1.9 | 1.9 |
| South Africa | 295,456 | 56,015 | 63,171 | - | - | - | 4,210 | 8,325 | 57.2 | 10.6 | 26.3 | 5.9 |
| South Sudan | 9,015 | 12,231 | 80 | 587 | 19 | 102 | 4 | 20 | 83.6 | 4.5 | 11.9 | 0.0 |

GDP

Pop.

Pubs. Cit.

F-a

F-a

2008

2017

Int.

Nat.

Inst.

Single

**Table S1:** Bibliometric data

Country

|  | GDP (million) | (thousand) |  |  | pubs. | cit. | pubs. | pubs. | Coll. | Coll. | Coll. | author |
| --- | --- | --- | --- | --- | --- | --- | --- | --- | --- | --- | --- | --- |
| Sudan | 95,584 | 39,579 | 3,620 | 46,793 | 1,949 | 10,599 | 214 | 442 | 76.2 | 0.1 | 20.1 | 3.6 |
| Swaziland | 3,721 | 1,343 | 542 | 5,871 | 199 | 1,727 | 31 | 92 | 86.8 | 0.7 | 7.9 | 4.6 |
| Tanzania | 47,340 | 55,572 | 7,716 | 119,936 | 3,167 | 26,771 | 429 | 1,061 | 83.2 | 1.4 | 13.3 | 2.0 |
| Togo | 4,400 | 7,606 | 924 | 6,331 | 562 | 1,739 | 51 | 107 | 65.2 | 0.0 | 34.5 | 0.3 |
| Tunisia | 42,063 | 11,403 | 20,532 | 182,898 | 17,138 | 117,263 | 1,592 | 2,373 | 47.6 | 7.8 | 43.5 | 1.1 |
| Uganda | 24,079 | 41,488 | 8,250 | 145,435 | 3,496 | 34,353 | 441 | 1,171 | 85.6 | 2.2 | 10.8 | 1.5 |
| Zambia | 21,064 | 16,591 | 2,758 | 49,329 | 861 | 8,047 | 218 | 446 | 90.8 | 2.2 | 5.5 | 1.5 |
| Zimbabwe | 16,620 | 16,150 | 3,005 | 45,515 | 1,320 | 9,604 | 205 | 469 | 75.2 | 1.8 | 19.6 | 3.4 |

Pop.

Pubs. Cit.

F-a

F-a

2008

2017

Int.

Nat.

Inst.

Single

Cit., citations; coll., collaboration; F-A, first-author; GDP, gross domestic product; inst., institutional; int., international; nat., national; pop., population; pubs., publications Collaboration figures are from SciVal and reflect outputs published in 2013-2017. All other data cover outputs published in 2008-2017.

# Table S2: Clinical trial infrastructures and intellectual property rights

| GDP  Country | | Pop. | Trials | Trials | Trials | Patent | Patent apps |
| --- | --- | --- | --- | --- | --- | --- | --- |
|  | (million) | (thousand) | (ICTRP) | per 1 mill. | (ct.gov) | apps | per 1 mill. |
| Algeria | 159,049 | 40,606 | 165 | 4.06 | 95 | 106 | 2.61 |
| Angola | 95,335 | 28,813 | 16 | 0.56 | 3 | - | - |
| Benin | 8,583 | 10,872 | 54 | 4.97 | 38 | - | - |
| Botswana | 15,581 | 2,250 | 93 | 41.33 | 64 | 1 | 0.44 |
| Burkina Faso | 11,693 | 18,646 | 186 | 9.98 | 126 | 2 | 0.11 |
| Burundi | 3,007 | 10,524 | 19 | 1.81 | 11 | - | - |
| Cameroon | 32,218 | 23,439 | 113 | 4.82 | 68 | - | - |
| Cape Verde | 1,617 | 540 | 0 | 0.00 | 0 | - | - |
| Central African Republic | 1,756 | 4,595 | 13 | 2.83 | 6 | - | - |
| Chad | 9,601 | 14,453 | 12 | 0.83 | 7 | - | - |
| Comoros | 617 | 796 | 3 | 3.77 | 1 | - | - |
| Democratic Republic of the Congo | 35,382 | 78,736 | 87 | 1.10 | 55 | - | - |
| Djibouti | 1,727 | 942 | 4 | 4.24 | 1 | 1 | 1.06 |
| Egypt | 332,791 | 95,689 | 3,711 | 38.78 | 2,409 | 918 | 9.59 |
| Equatorial Guinea | 10,685 | 1,221 | 6 | 4.91 | 3 | - | - |
| Eritrea | 2,608 | 4,475 | 6 | 1.34 | 0 | - | - |
| Ethiopia | 72,374 | 102,403 | 188 | 1.84 | 124 | 12 | 0.12 |
| Gabon | 14,214 | 1,980 | 63 | 31.82 | 44 | - | - |
| Gambia | 965 | 2,039 | 119 | 58.38 | 67 | - | - |
| Ghana | 42,690 | 28,207 | 238 | 8.44 | 137 | 14 | 0.50 |
| Guinea | 8,200 | 12,396 | 31 | 2.50 | 20 | - | - |
| Guinea-Bissau | 1,165 | 1,816 | 62 | 34.15 | 51 | - | - |
| Ivory Coast | 36,373 | 23,696 | 72 | 3.04 | 39 | 26 | 1.10 |

**Table S2:** Clinical trial infrastructures and intellectual property rights

| GDP  Country | | Pop. | Trials | Trials | Trials | Patent | Patent apps |
| --- | --- | --- | --- | --- | --- | --- | --- |
|  | (million) | (thousand) | (ICTRP) | per 1 mill. | (ct.gov) | apps | per 1 mill. |
| Kenya | 70,529 | 48,462 | 639 | 13.19 | 417 | 144 | 2.97 |
| Lesotho | 2,291 | 2,204 | 22 | 9.98 | 16 | - | - |
| Liberia | 2,101 | 4,614 | 25 | 5.42 | 16 | - | - |
| Libya | 34,699 | 6,293 | 19 | 3.02 | 6 | - | - |
| Madagascar | 10,001 | 24,895 | 27 | 1.08 | 15 | 6 | 0.24 |
| Malawi | 5,433 | 18,092 | 317 | 17.52 | 201 | 3 | 0.17 |
| Mali | 14,035 | 17,995 | 170 | 9.45 | 128 | - | - |
| Mauritania | 4,739 | 4,301 | 10 | 2.33 | 1 | - | - |
| Mauritius | 12,168 | 1,263 | 34 | 26.91 | 20 | 2 | 1.58 |
| Morocco | 103,606 | 35,277 | 191 | 5.41 | 96 | 237 | 6.72 |
| Mozambique | 11,015 | 28,829 | 97 | 3.36 | 68 | 15 | 0.52 |
| Namibia | 10,948 | 2,480 | 7 | 2.82 | 3 | - | - |
| Niger | 7,528 | 20,673 | 34 | 1.64 | 26 | - | - |
| Nigeria | 404,653 | 185,990 | 342 | 1.84 | 139 | 50 | 0.27 |
| Republic of the Congo | 7,834 | 5,126 | 52 | 10.14 | 37 | - | - |
| Rwanda | 8,376 | 11,918 | 97 | 8.14 | 71 | 2 | 0.17 |
| Sao Tome and Principe | 343 | 200 | 2 | 10.00 | 0 | - | - |
| Senegal | 14,684 | 15,412 | 103 | 6.68 | 75 | - | - |
| Seychelles | 1,427 | 95 | 3 | 31.69 | 1 | - | - |
| Sierra Leone | 3,737 | 7,396 | 39 | 5.27 | 17 | - | - |
| Somalia | 6,217 | 14,318 | 14 | 0.98 | 0 | - | - |
| South Africa | 295,456 | 56,015 | 4,341 | 77.50 | 2,505 | 2,783 | 49.68 |
| South Sudan | 9,015 | 12,231 | 2 | 0.16 | 0 | - | - |

**Table S2:** Clinical trial infrastructures and intellectual property rights

| GDP  Country | | Pop. | Trials | Trials | Trials | Patent | Patent apps |
| --- | --- | --- | --- | --- | --- | --- | --- |
|  | (million) | (thousand) | (ICTRP) | per 1 mill. | (ct.gov) | apps | per 1 mill. |
| Sudan | 95,584 | 39,579 | 71 | 1.79 | 34 | 284 | 7.18 |
| Swaziland | 3,721 | 1,343 | 22 | 16.38 | 13 | - | - |
| Tanzania | 47,340 | 55,572 | 433 | 7.79 | 290 | 1 | 0.02 |
| Togo | 4,400 | 7,606 | 16 | 2.10 | 8 | - | - |
| Tunisia | 42,063 | 11,403 | 426 | 37.36 | 255 | 235 | 20.61 |
| Uganda | 24,079 | 41,488 | 651 | 15.69 | 447 | 16 | 0.39 |
| Zambia | 21,064 | 16,591 | 262 | 15.79 | 171 | 14 | 0.84 |
| Zimbabwe | 16,620 | 16,150 | 190 | 11.76 | 111 | 8 | 0.50 |

Apps, applications; GDP, gross domestic product; ICTRP, International Clinical Trials Registry Platform.

# Table S3: R&D personnel

| GDP  Country | | Pop. | R&D staff | Researchers | Researchers | Researchers |
| --- | --- | --- | --- | --- | --- | --- |
|  | (million) | (thousand) | per mill. | per mill. | (% M&HS) | (% PhD) |
| Algeria | 159,049 | 40,606 | 220.23 | 168.02 | 6.87 | 16.22 |
| Angola | 95,335 | 28,813 | 84.15 | 47.48 |  | 20.09 |
| Benin | 8,583 | 10,872 | - | - | - | - |
| Botswana | 15,581 | 2,250 | 570.35 | 179.47 | 18.31 | 29.37 |
| Burkina Faso | 11,693 | 18,646 | 131.33 | 47.58 | 46.37 | 41.85 |
| Burundi | 3,007 | 10,524 | - | - | - | - |
| Cameroon | 32,218 | 23,439 | - | - | - | - |
| Cape Verde | 1,617 | 540 | 72.83 | 49.21 | 0.00 | 72.00 |
| Central African Republic | 1,756 | 4,595 | - | - | - | - |
| Chad | 9,601 | 14,453 | 75.90 | 58.33 | 6.49 | 29.72 |
| Comoros | 617 | 796 | - | - | - | - |
| Democratic Republic of the Congo | 35,382 | 78,736 | 19.45 | 7.23 | 7.68 | 11.95 |
| Djibouti | 1,727 | 942 | - | - | - | - |
| Egypt | 332,791 | 95,689 | 1,208.51 | 680.30 | 29.80 | 55.86 |
| Equatorial Guinea | 10,685 | 1,221 | - | - | - | - |
| Eritrea | 2,608 | 4,475 | - | - | - | - |
| Ethiopia | 72,374 | 102,403 | 121.21 | 44.97 | 12.98 | 16.66 |
| Gabon | 14,214 | 1,980 | - | - | - | - |
| Gambia | 965 | 2,039 | 603.31 | 33.56 | 40.96 | 55.63 |
| Ghana | 42,690 | 28,207 | 122.57 | 38.37 | 14.40 | 34.39 |
| Guinea | 8,200 | 12,396 | - | - | - | - |
| Guinea-Bissau | 1,165 | 1,816 | - | - | - | - |
| Ivory Coast | 36,373 | 23,696 | - | 69.21 | - | - |

**Table S3:** R&D personnel

| Country GDP | | Pop. | R&D staff | Researchers | Researchers | Researchers |
| --- | --- | --- | --- | --- | --- | --- |
|  | (million) | (thousand) | per mill. | per mill. | (% M&HS) | (% PhD) |
| Kenya | 70,529 | 48,462 | 1,029.40 | 225.03 | 25.82 | 6.07 |
| Lesotho | 2,291 | 2,204 | 32.90 | 22.83 | 0.00 | 28.06 |
| Liberia | 2,101 | 4,614 | - | - | - | - |
| Libya | 34,699 | 6,293 | - | - | - | - |
| Madagascar | 10,001 | 24,895 | 113.00 | 24.70 | 13.17 | 46.99 |
| Malawi | 5,433 | 18,092 | 113.44 | 48.27 | 18.99 | 9.89 |
| Mali | 14,035 | 17,995 | 73.45 | 30.79 | 11.25 | 61.79 |
| Mauritania | 4,739 | 4,301 | - | - | - | - |
| Mauritius | 12,168 | 1,263 | 500.18 | 181.83 | 6.59 | 27.58 |
| Morocco | 103,606 | 35,277 | 1,149.30 | 1,068.96 | 9.22 | 16.24 |
| Mozambique | 11,015 | 28,829 | 82.83 | 41.48 | 11.65 | 13.99 |
| Namibia | 10,948 | 2,480 | 235.68 | 143.32 | 3.47 | 21.76 |
| Niger | 7,528 | 20,673 | 43.69 | 7.42 | - | - |
| Nigeria | 404,653 | 185,990 | 77.38 | 38.77 | - | 34.11 |
| Republic of the Congo | 7,834 | 5,126 | 67.27 | 31.54 | - | - |
| Rwanda | 8,376 | 11,918 | - | 12.35 | - | 37.66 |
| Sao Tome and Principe | 343 | 200 | - | - | - | - |
| Senegal | 14,684 | 15,412 | 622.86 | 549.32 | 14.07 | 38.68 |
| Seychelles | 1,427 | 95 | 2,028.31 | 146.49 |  | 15.38 |
| Sierra Leone | 3,737 | 7,396 | - | - | - | - |
| Somalia | 6,217 | 14,318 | - | - | - | - |
| South Africa | 295,456 | 56,015 | 742.51 | 473.12 |  | 35.22 |
| South Sudan | 9,015 | 12,231 | - | - | - | - |

**Table S3:** R&D personnel

| Country GDP | | Pop. | R&D staff | Researchers | Researchers | Researchers |
| --- | --- | --- | --- | --- | --- | --- |
|  | |  |  |  |  |  |
|  | (million) | (thousand) | per mill. | per mill. | (% M&HS) | (% PhD) |
| Sudan | 95,584 | 39,579 | - | - | - | - |
| Swaziland | 3,721 | 1,343 | 308.97 | 119.14 | 33.93 | 24.98 |
| Tanzania | 47,340 | 55,572 | 38.84 | 18.34 | - | 32.78 |
| Togo | 4,400 | 7,606 | 42.42 | 31.77 | 16.11 | 68.88 |
| Tunisia | 42,063 | 11,403 | 2,068.71 | 1,964.97 | - | 35.07 |
| Uganda | 24,079 | 41,488 | 41.54 | 26.47 | 19.55 | 30.51 |
| Zambia | 21,064 | 16,591 | 162.81 | 40.97 | - | - |
| Zimbabwe | 16,620 | 16,150 | 118.34 | 88.72 | 0.23 | 13.45 |

GDP, gross domestic product; M&HS, medical and health sciences; mill., million; pop., population; PhD, doctor of philosophy; R&D, research and development. All data are from 2016 or most recent year available.

# Table S4: R&D expenditure

Country

|  | (million) | (thousand) |  | (% GDP) | per capita | (M&HS) | per researcher | (M&HS %) |
| --- | --- | --- | --- | --- | --- | --- | --- | --- |
| Algeria | 159,049 | 40,606 | 241,204.78 | 0.07 | 7.25 | - | 43.13 | - |
| Angola | 95,335 | 28,813 | - | - | - | - | - | - |
| Benin | 8,583 | 10,872 | - | - | - | - | - | - |
| Botswana | 15,581 | 2,250 | 184,247.17 | 0.54 | 86.56 | 23,143.69 | 482.32 | 30.04 |
| Burkina Faso | 11,693 | 18,646 | 65,025.67 | 0.22 | 3.70 | 2,910.88 | - | 4.48 |
| Burundi | 3,007 | 10,524 | 8,460.33 | 0.12 | 0.94 | - | - | - |
| Cameroon | 32,218 | 23,439 | - | - | - | - | - | - |
| Cape Verde | 1,617 | 540 | 2,211.43 | 0.07 | 4.35 |  | 88.46 | - |
| Central African Republic | 1,756 | 4,595 | - | - | - | - | - | - |
| Chad | 9,601 | 14,453 | 91,046.10 | 0.32 | 6.30 | 14,722.82 | 108.00 | 16.17 |
| Comoros | 617 | 796 | - | - | - | - | - | - |
| Democratic Republic of the Congo | 35,382 | 78,736 | 10,234.03 | 0.02 | 0.13 | - | 18.58 | - |
| Djibouti | 1,727 | 942 | - | - | - | - | - | - |
| Egypt | 332,791 | 95,689 | 7,562,293.01 | 0.71 | 79.03 | - | 116.17 | - |
| Equatorial Guinea | 10,685 | 1,221 | - | - | - | - | - | - |
| Eritrea | 2,608 | 4,475 | - | - | - | - | - | - |
| Ethiopia | 72,374 | 102,403 | 787,274.45 | 0.60 | 8.30 | 34,522.66 | 184.52 | 15.50 |
| Gabon | 14,214 | 1,980 | 131,865.44 | 0.58 | 83.10 | - | - | - |
| Gambia | 965 | 2,039 | 3,543.77 | 0.13 | 2.03 | - | 60.47 | - |
| Ghana | 42,690 | 28,207 | 276,671.79 | 0.38 | 11.29 | - | 294.14 | - |
| Guinea | 8,200 | 12,396 | - | - | - | - | - | - |
| Guinea-Bissau | 1,165 | 1,816 | - | - | - | - | - | - |
| Ivory Coast | 36,373 | 23,696 | - | - | - | - | - | - |

GDP

Pop.

GERD

GERD

GERD

GERD

GERD

GERD

**Table S4:** R&D expenditure

Country

|  | (million) | (thousand) |  | (% GDP) | per capita | (M&HS) | per researcher | (M&HS %) |
| --- | --- | --- | --- | --- | --- | --- | --- | --- |
| Kenya | 70,529 | 48,462 | 788,176.89 | 0.79 | 19.06 | 216,530.58 | 84.70 | 27.47 |
| Lesotho | 2,291 | 2,204 | 3,018.33 | 0.05 | 1.39 | 0.00 | 60.79 | 0.00 |
| Liberia | 2,101 | 4,614 | - | - | - | - | - | - |
| Libya | 34,699 | 6,293 | - | - | - | - | - | - |
| Madagascar | 10,001 | 24,895 | 5,626.29 | 0.01 | 0.23 | 768.68 | 9.15 | 13.66 |
| Malawi | 5,433 | 18,092 |  |  |  |  |  |  |
| Mali | 14,035 | 17,995 | 112,531.28 | 0.31 | 6.44 | 9,256.23 | 209.22 | 8.23 |
| Mauritania | 4,739 | 4,301 | - | - | - | - | - | - |
| Mauritius | 12,168 | 1,263 | 38,856.48 | 0.18 | 31.00 | 1,714.62 | 170.50 | 4.41 |
| Morocco | 103,606 | 35,277 | 1,483,610.29 | 0.71 | 45.78 |  | 63.73 |  |
| Mozambique | 11,015 | 28,829 | 112,790.11 | 0.34 | 4.03 | 32,234.22 | 97.08 | 28.58 |
| Namibia | 10,948 | 2,480 | 81,658.45 | 0.34 | 34.44 | 1,585.30 | 240.31 | 1.94 |
| Niger | 7,528 | 20,673 | - | - | - | - | - | - |
| Nigeria | 404,653 | 185,990 | 1,374,848.32 | 0.22 | 9.39 | 142,133.56 | 242.20 | 10.34 |
| Republic of the Congo | 7,834 | 5,126 | - | - | - | - | - | - |
| Rwanda | 8,376 | 11,918 | - | - | - | - | - | - |
| Sao Tome and Principe | 343 | 200 | - | - | - | - | - | - |
| Senegal | 14,684 | 15,412 | 275,579.68 | 0.75 | 18.40 | - | 33.50 | - |
| Seychelles | 1,427 | 95 | 5,961.26 | 0.22 | 63.26 | - | 305.37 | - |
| Sierra Leone | 3,737 | 7,396 | - | - | - | - | - | - |
| Somalia | 6,217 | 14,318 | - | - | - | - | - | - |
| South Africa | 295,456 | 56,015 | 5,823,288.67 | 0.80 | 105.32 | 905,563.36 | 222.61 | 18.19 |
| South Sudan | 9,015 | 12,231 | - | - | - | - | - | - |

GDP

Pop.

GERD

GERD

GERD

GERD

GERD

GERD

**Table S4:** R&D expenditure

Country

|  | (million) | (thousand) |  | (% GDP) | per capita | (M&HS) | per researcher | (M&HS %) |
| --- | --- | --- | --- | --- | --- | --- | --- | --- |
| Sudan | 95,584 | 39,579 | 281,296.26 | 0.30 | 9.10 | - | - | - |
| Swaziland | 3,721 | 1,343 | 29,493.90 | 0.27 | 22.36 | 8,923.15 | 187.68 | 30.25 |
| Tanzania | 47,340 | 55,572 | 623,754.21 | 0.53 | 12.32 | - | 671.75 | - |
| Togo | 4,400 | 7,606 | 27,209.82 | 0.27 | 3.76 | 4,317.74 | 100.19 | 15.87 |
| Tunisia | 42,063 | 11,403 | 794,749.43 | 0.60 | 69.70 | - | 35.47 | - |
| Uganda | 24,079 | 41,488 | 114,149.62 | 0.17 | 2.94 | 14,811.03 | 111.06 | 12.98 |
| Zambia | 21,064 | 16,591 | 100,756.29 | 0.28 | 7.70 | - | 187.98 | - |
| Zimbabwe | 16,620 | 16,150 | - | - | - | - | - | - |

GDP

Pop.

GERD

GERD

GERD

GERD

GERD

GERD

GERD, gross expenditure on research and development; GDP, gross domestic product; M&HS, medical and health sciences; pop., population; PhD, doctor of philosophy; R&D, research and development.

All data are from 2016 or most recent year available.

# Table S5: Regulatory capacities

Country

|  | (million) | (thousand) |  | | | | | | | |
| --- | --- | --- | --- | --- | --- | --- | --- | --- | --- | --- |
| Algeria | 159,049 | 40,606 | 1 | 0 | 2 | 0 | - | Y | 1 | |
| Angola | 95,335 | 28,813 | - | - | - | - | Y | - | 0 | |
| Benin | 8,583 | 10,872 | 0 | 1 | 0 | 0 | - | Y | 3 | |
| Botswana | 15,581 | 2,250 | 3 | 2 | 1 | 5 | - | Y | 4 | |
| Burkina Faso | 11,693 | 18,646 | 1 | 0 | 2 | 0 | - | Y | 4 | |
| Burundi | 3,007 | 10,524 | - | - | - | - | Y | - | 0 | |
| Cameroon | 32,218 | 23,439 | 1 | 0 | 1 | 1 | Y | - | 8 | |
| Cape Verde | 1,617 | 540 | - | - | - | - | Y | Y | 0 | |
| Central African Republic | 1,756 | 4,595 | - | - | - | - | - | - | 1 | |
| Chad | 9,601 | 14,453 | - | - | - | - | - | - | 0 | |
| Comoros | 617 | 796 | - | - | - | - | - | - | 0 | |
| Democratic Republic of the Congo | 35,382 | 78,736 | 0 | 1 | 0 | 0 | - | Y | 4 | |
| Djibouti | 1,727 | 942 | - | - | - | - | - | - | 0 | |
| Egypt | 332,791 | 95,689 | 2 | 1 | 1 | 0 | - | Y | | 23 |
| Equatorial Guinea | 10,685 | 1,221 | - | - | - | - | - | - | 0 | |
| Eritrea | 2,608 | 4,475 | - | - | - | - | - | - | 0 | |
| Ethiopia | 72,374 | 102,403 | 3 | 1 | 1 | 2 | Y | Y | 7 | |
| Gabon | 14,214 | 1,980 | - | - | - | - | - | Y | 1 | |
| Gambia | 965 | 2,039 | 1 | 0 | 0 | 1 | - | Y | 1 | |
| Ghana | 42,690 | 28,207 | 1 | 1 | 1 | 4 | Y | Y | 3 | |
| Guinea | 8,200 | 12,396 | 2 | 2 | 1 | 1 | Y | - | 0 | |
| Guinea-Bissau | 1,165 | 1,816 | - | - | - | - | Y | - | 0 | |
| Ivory Coast | 36,373 | 23,696 | 1 | 0 | 1 | 0 | Y | Y | 1 | |

GDP

Pop.

Organizations Legislation Regulations Guidelines NPHI NEC IRBs

**Table S5:** Regulatory capacities

Country

|  | (million) | (thousand) |  | | | | | | |
| --- | --- | --- | --- | --- | --- | --- | --- | --- | --- |
| Kenya | 70,529 | 48,462 | 4 | 3 | 2 | 2 | Y | - | 4 |
| Lesotho | 2,291 | 2,204 | - | - | - | - | - | - | 1 |
| Liberia | 2,101 | 4,614 | 2 | 0 | 2 | 1 | Y | - | 2 |
| Libya | 34,699 | 6,293 | - | - | - | - | Y | - | 1 |
| Madagascar | 10,001 | 24,895 | 0 | 1 | 0 | 0 | Y | Y | 2 |
| Malawi | 5,433 | 18,092 | 8 | 5 | 2 | 8 | Y | Y | 2 |
| Mali | 14,035 | 17,995 | 1 | 1 | 0 | 0 | - | Y | 1 |
| Mauritania | 4,739 | 4,301 | - | - | - | - | - | - | 0 |
| Mauritius | 12,168 | 1,263 | - | - | - | - | - | Y | 2 |
| Morocco | 103,606 | 35,277 | - | - | - | - | Y | - | 2 |
| Mozambique | 11,015 | 28,829 | 0 | 0 | 0 | 1 | Y | - | 0 |
| Namibia | 10,948 | 2,480 | - | - | - | - | - | - | 1 |
| Niger | 7,528 | 20,673 | - | - | - | - | - | - | 1 |
| Nigeria | 404,653 | 185,990 | 5 | 2 | 0 | 6 | Y | Y | 26 |
| Republic of the Congo | 7,834 | 5,126 | - | - | - | - | - | Y | 2 |
| Rwanda | 8,376 | 11,918 | 1 | 0 | 0 | 1 | Y | Y | 3 |
| Sao Tome and Principe | 343 | 200 | - | - | - | - | Y | - | 0 |
| Senegal | 14,684 | 15,412 | 1 | 1 | 0 | 0 | - | Y | 2 |
| Seychelles | 1,427 | 95 | - | - | - | - | - | - | 0 |
| Sierra Leone | 3,737 | 7,396 | 3 | 0 | 3 | 3 | Y | - | 0 |
| Somalia | 6,217 | 14,318 | - | - | - | - | Y | - | 0 |
| South Africa | 295,456 | 56,015 | 11 | 5 | 7 | 9 | Y | Y | 30 |
| South Sudan | 9,015 | 12,231 | - | - | - | - | - | - | 0 |

GDP

Pop.

Organizations Legislation Regulations Guidelines NPHI NEC IRBs

**Table S5:** Regulatory capacities

Country

|  | (million) | (thousand) |  | | | | | | |
| --- | --- | --- | --- | --- | --- | --- | --- | --- | --- |
| Sudan | 95,584 | 39,579 | 5 | 3 | 0 | 4 | Y | Y | 7 |
| Swaziland | 3,721 | 1,343 | - | - | - | - | - | - | 0 |
| Tanzania | 47,340 | 55,572 | 5 | 5 | 3 | 3 | Y | - | 5 |
| Togo | 4,400 | 7,606 | 2 | 0 | 1 | 2 | Y | Y | 1 |
| Tunisia | 42,063 | 11,403 | - | - | - | - | - | Y | 2 |
| Uganda | 24,079 | 41,488 | 2 | 2 | 0 | 1 | Y | Y | 9 |
| Zambia | 21,064 | 16,591 | 2 | 3 | 0 | 1 | Y | - | 3 |
| Zimbabwe | 16,620 | 16,150 | 5 | 6 | 3 | 4 | - | - | 3 |

GDP

Pop.

Organizations Legislation Regulations Guidelines NPHI NEC IRBs

GDP, gross domestic product; IRB, institutional review board; NEC, national ethics committee NPHI, national public health institute; pop., population.

# Table S6: Funding

| Country | WT | MRC | NIH | CIHR | NHMRC | INSERM | DFG | HHMI |  | EC | CDMRP |
| --- | --- | --- | --- | --- | --- | --- | --- | --- | --- | --- | --- |
| Algeria | - | - |  | - | - | - | - | - |  | 1,568,955.24 | - |
| Benin | - | - | 1,446,653.00 | - | - | - | - | - |  | - | - |
| Botswana | 184,186.00 | - | 7,356,998.00 | - | - | - | - | - |  | - | - |
| Burkina Faso | 39,300.00 | - |  | - | - | - | - | - |  | - | - |
| Cameroon | 133,593.80 | - |  | - | - | - | - | - | 222 | 2,315,190.99 | - |
| Ivory Coast | 9,177,381.85 | - |  | - | - | - | - | - |  | - | - |
| Democratic Republic of the Congo | 0.00 | - | 451,242.00 | - | - | - | - | - |  | - | - |
| Egypt | 0.00 | - | 384,112.00 | - | - | - | - | - |  | 5,710,715.90 | - |
| Ethiopia | 0.00 | 156,726.38 | 4,045,740.00 | - | - | - | - | - |  | - | - |
| Gambia | 2,201,155.01 | 93,350,867.24 | 220,300.00 | - | - | - | - | - |  | - | - |
| Ghana | 6,802,150.11 | - | 19,915,940.00 | - | - | - | - | - |  | 17,641.05 | - |
| Kenya | 120,959,548.23 | 762,618.20 | 23,965,606.00 | - | - | - | - | - |  | 2,532,293.26 | - |
| Libya | - | - | - | - | - | - | - | - |  | 694,291.15 |  |
| Madagascar | 0.00 | - | 269,867.00 | - | - | - | - | - |  | - | - |
| Malawi | 8,620,144.53 | - | 3,239,240.00 | - | - | - | - | - |  | - | - |
| Mali | 1,317,039.94 | - | 7,315,257.00 | - | - | - | - | - |  | - | - |
| Morocco | - | - | - | - | - | - | - | - | - | 3,357,582.35 |  |
| Mozambique | 0.00 | - | 14,014,017.00 | - | - | - | - | - |  | - | - |
| Namibia | 0.00 | - |  | - | - | - | - | - |  | - | - |
| Nigeria | 1,002,984.47 | - | 34,273,898.00 | - | - | - | - | - |  | 17,641.05 | - |
| Rwanda | 0.00 | - | 1,539,861.00 | - | - | - | - | - |  | - | - |
| Senegal | 1,373,601.81 | 1,720,283.81 | - | - | - | - | - | - |  | 21,006.48  2,513,725 | - |
| South Africa | 77,994,865.15 | 13,302,915.07 | 388,950,849.00 | - | - | - | - | - |  | 2,513,725.77 | - |
| Sudan | 0.00 | - | - | - | - | - | - | - |  | - | - |
| Tanzania | 16,673,318.44 | - | 4,764,355.00 | - | - | - | - | - |  | - | - |
| Tunisia | 0.00 | - | 2,178,511.00 | - | - | - | - | - |  | 7,307,461.63 | - |
| Uganda | 17,943,085.72 | 40,607,133.72 | 35,999,665.00 | - | - | - | - | - |  | 17,641.05 | - |

**Table S6:** Funding

| Country | WT | MRC | NIH | CIHR | NHMRC | INSERM | DFG | HHMI | EC | CDMRP |
| --- | --- | --- | --- | --- | --- | --- | --- | --- | --- | --- |
| Zambia | 15,720.00 | - | 13,073,179.00 | - | - | - | - | - | - | - |
| Zimbabwe | 1,389,486.87 | - | 21,663,497.00 | - | - | - | - | - | - | - |

CIHR, Canadian Institutes of Health Research; CDMRP, Congressional Directed Medical Research Programs (Department of Defence); DFG, German Research Foundation (Deutsche Forschungsgemeinschaft); EC, European Commission; HHMI, Howard Hughes Medical Institute; INSERM, Institut national de la santé et de la recherche médicale; MRC, Medical Research Council; NHMRC, National health and Medical Research Council; NIH, National Institutes of Health; WT, Wellcome Trust

*Cells for CIHR, NHMRC, INSERM, HHMI, CDMRP, DFG are empty because data did not meet inclusion criteria. We excluded funding for research projects in which the principal investigators were based at non-African institutions, even if these projects included collaborators, field sites, or locations of research in Africa.


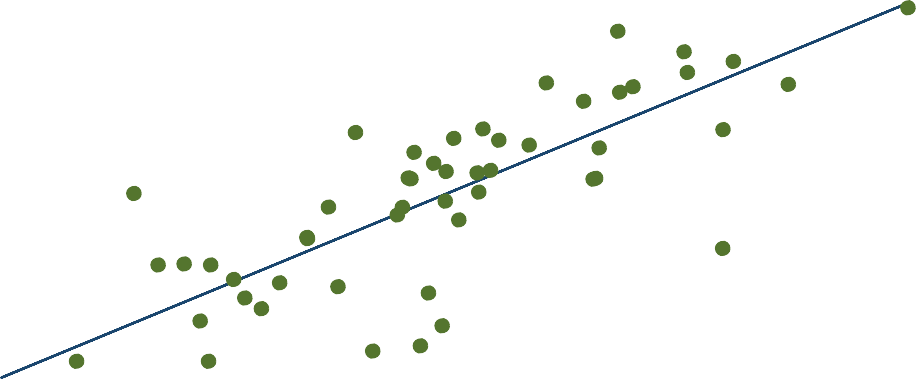


6

8

10

gdp_ln

12

14

95% CI

pub_ln

Fitted values

2

4

6

8

10

12

**Figure S1. The relationship between gross domestic product and publications**

**Note**: Both variables are expressed as natural logs.

**Table S7.** Regression summary for gross domestic product and number of publications

(1)

pub_ln

gdp_ln 0.934∗∗∗

(10.52)

Constant −1.630

(−1.93)

Observations 54

*R*2 0.680

Adjusted *R*2 0.674

*t* statistics in parentheses

∗ *p* < 0.05, ∗∗ *p* < 0.01, ∗∗∗ *p* < 0.001


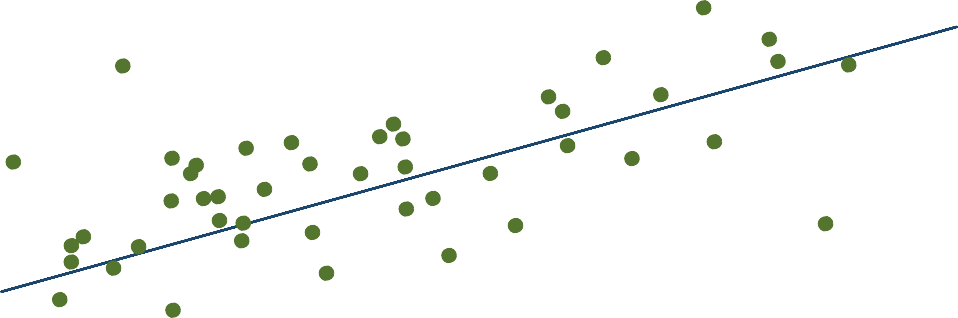


6

7

8

gdp_cap_ln

9

10

95% CI

pub_cap_ln

Fitted values

−12

−10

−8

−6

**Figure S2. The relationship between gross domestic product per capita and publications per capita**

**Note**: Both variables are expressed as natural logs.

**Table S8.** Regression summary for gross domestic product per capita and the number of publications per capita

(1)

pub_cap_ln

gdp_cap_ln 0.771∗∗∗

(5.56)

Constant −14.42∗∗∗

(−14.39)

Observations 54

*R*2 0.373

Adjusted *R*2 0.361

*t* statistics in parentheses

∗ *p* < 0.05, ∗∗ *p* < 0.01, ∗∗∗ *p* < 0.001


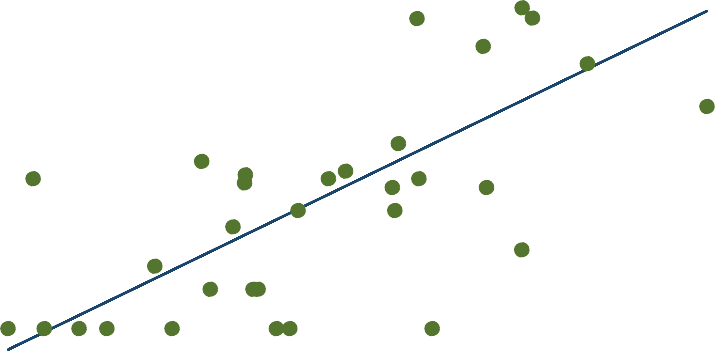


6

8

10

gdp_ln

12

14

95% CI

patent_ln

Fitted values

0

2

4

6

8

Figure S3. The relationship between gross domestic product and patent applications

−2

**Note**: Both variables are expressed as natural logs.

**Table S9.** Regression summary for gross domestic product and patent applications

(1)

patent_ln

gdp_ln 1.093∗∗∗

(6.27)

Constant −8.519∗∗∗

(−4.83)

Observations 34

*R*2 0.551

Adjusted *R*2 0.537

*t* statistics in parentheses

∗ *p* < 0.05, ∗∗ *p* < 0.01, ∗∗∗ *p* < 0.001


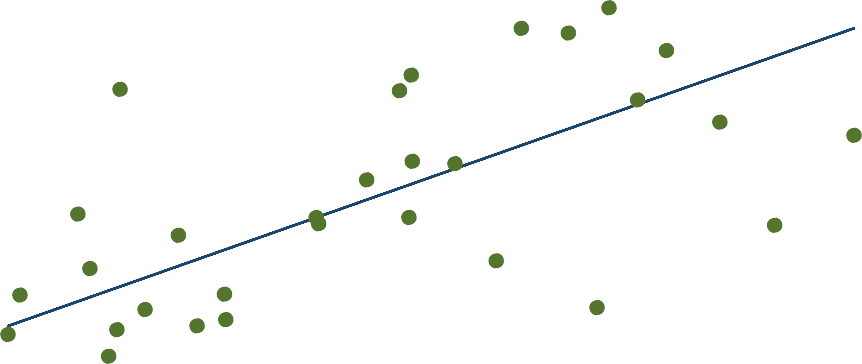


6

7

8

gdp_cap_ln

9

10

95% CI

patent_mill_ln

Fitted values

−2

0

2

4

**Figure S4. The relationship between gross domestic product per capita and patent applications per capita**

−4

**Note**: Both variables are expressed as natural logs.

**Table S10.** Regression summary for gross domestic product per capita and patent applications per capita

(1)

patent_mill_ln

gdp_cap_ln 1.196∗∗∗

(4.65)

Constant −8.996∗∗∗

(−4.81)

Observations 34

*R*2 0.403

Adjusted *R*2 0.384

*t* statistics in parentheses

∗ *p* < 0.05, ∗∗ *p* < 0.01, ∗∗∗ *p* < 0.001


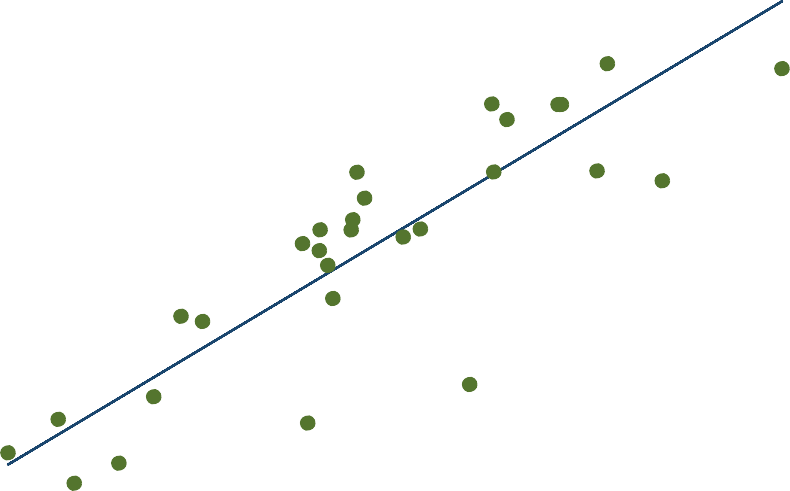


6

8

10

gdp_ln

12

14

95% CI

gerd_ln

Fitted values

8

10

12

14

16

Figure S5. The relationship between gross domestic product and GERD

**Note**: Both variables are expressed as natural logs.

**Table S11.** Regression summary for gross domestic product and GERD

(1)

gerd_ln

gdp_ln 1.190∗∗∗

(10.38)

Constant −0.188

(−0.16)

Observations 31

*R*2 0.788

Adjusted *R*2 0.781

*t* statistics in parentheses

∗ *p* < 0.05, ∗∗ *p* < 0.01, ∗∗∗ *p* < 0.001


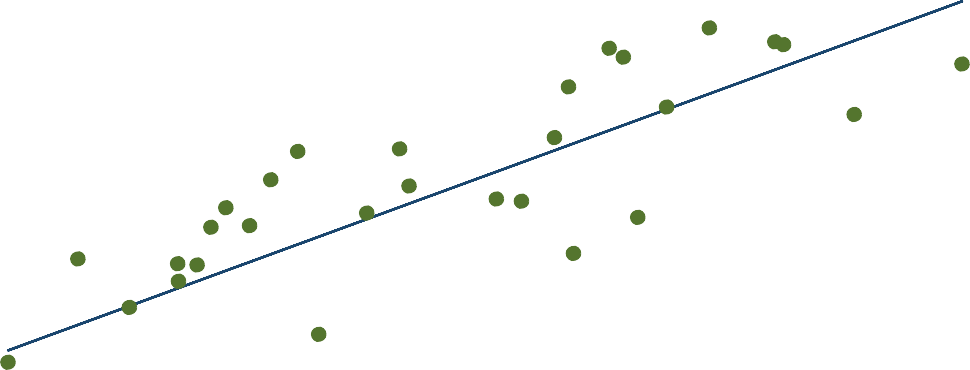


6

7

8

gdp_cap_ln

9

10

95% CI

gerd_cap_ln

Fitted values

0

2

4

6

**Figure S6. The relationship between gross domestic product per capita and GERD per capita**

−2

**Note**: Both variables are expressed as natural logs.

**Table S12.** Regression summary for gross domestic product per capita and GERD per capita

(1)

gerd_cap_ln

gdp_cap_ln 1.244∗∗∗

(7.17)

Constant −6.935∗∗∗

(−5.37)

Observations 31

*R*2 0.639

Adjusted *R*2 0.627

*t* statistics in parentheses

∗ *p* < 0.05, ∗∗ *p* < 0.01, ∗∗∗ *p* < 0.001


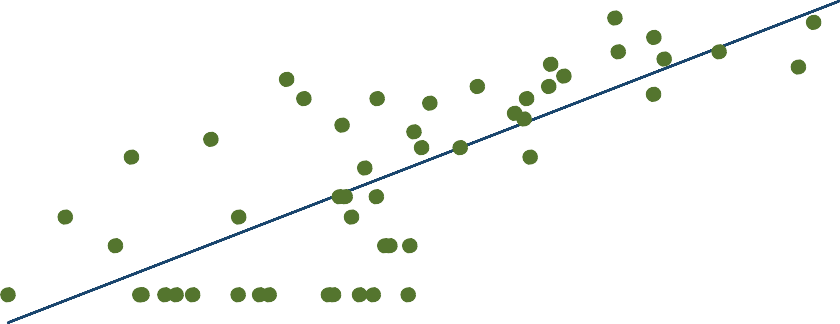


6

8

10

gdp_ln

12

14

95% CI

univ_ln

Fitted values

0

2

4

6

**Figure S7. The relationship between gross domestic product and universities**

−2

**Note**: Both variables are expressed as natural logs.

**Table S13.** Regression summary for gross domestic product and universities

(1)

univ_ln

gdp_ln 0.700∗∗∗

(8.55)

Constant −4.893∗∗∗

(−6.22)

Observations 52

*R*2 0.594

Adjusted *R*2 0.586

*t* statistics in parentheses

∗ *p* < 0.05, ∗∗ *p* < 0.01, ∗∗∗ *p* < 0.001


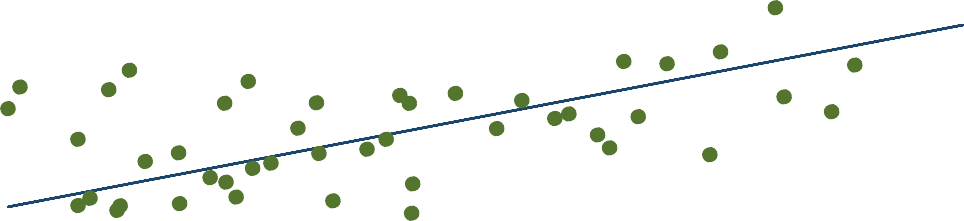


6

7

8

gdp_cap_ln

9

10

95% CI

univ_mill_ln

Fitted values

−2

0

2

4

**Figure S8. The relationship between gross domestic product per capita and universities per capita**

−4

**Note**: Both variables are expressed as natural logs.

**Table S14.** Regression summary for gross domestic product per capita and universities per capita

(1)

univ_mill_ln

gdp_cap_ln 0.648∗∗∗

(5.06)

Constant −5.207∗∗∗

(−5.62)

Observations 52

*R*2 0.339

Adjusted *R*2 0.326

*t* statistics in parentheses

∗ *p* < 0.05, ∗∗ *p* < 0.01, ∗∗∗ *p* < 0.001


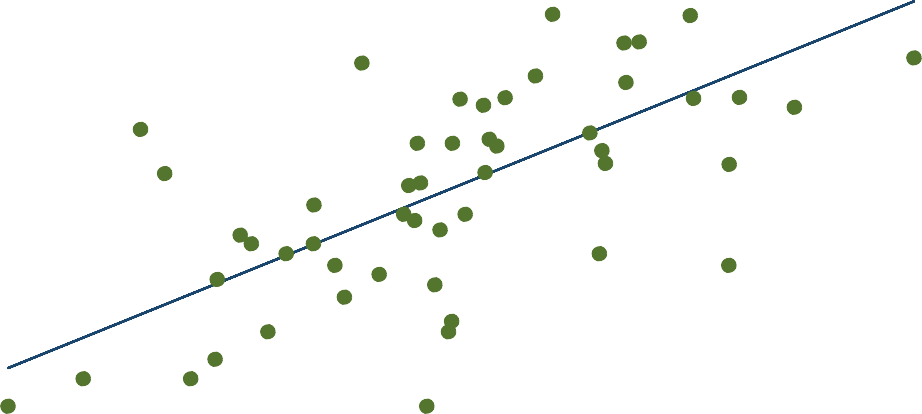


6

8

10

gdp_ln

12

14

95% CI

ictrp_ln

Fitted values

0

2

4

6

8

**Figure S9. The relationship between gross domestic product and clinical trials**

**Note**: Both variables are expressed as natural logs.

**Table S15.** Regression summary for gross domestic product and clinical trials

(1)

ictrp_ln

gdp_ln 0.765∗∗∗

(7.03)

Constant −3.212∗∗

(−3.09)

Observations 53

*R*2 0.492

Adjusted *R*2 0.482

*t* statistics in parentheses

∗ *p* < 0.05, ∗∗ *p* < 0.01, ∗∗∗ *p* < 0.001

0

2

4


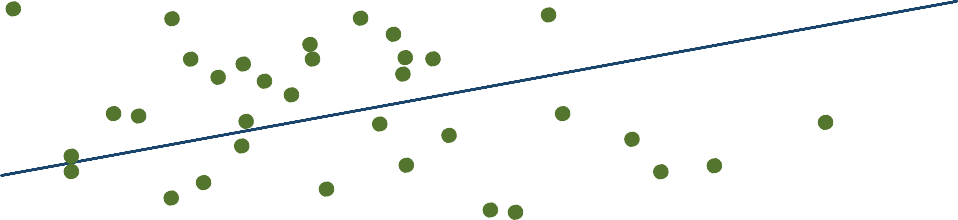


6

7

8

gdp_cap_ln

9

10

95% CI

ictrp_mill_ln

Fitted values

**Figure S10. The relationship between gross domestic product per capita and clinical trials per capita**

−2

**Note**: Both variables are expressed as natural logs.

**Table S16.** Regression summary for gross domestic product per capita and clinical trials per capita

(1)

ictrp_mill_ln

gdp_cap_ln 0.492∗∗

(2.98)

Constant −1.789

(-1.50)

Observations 53

*R*2 0.149

Adjusted *R*2 0.132

*t* statistics in parentheses

∗ *p* < 0.05, ∗∗ *p* < 0.01, ∗∗∗ *p* < 0.001
