# Supplementary material for: Measuring health science research and development in Africa: mapping the available data
Source: Health Res Policy Syst. 2021 Dec 11;19:142. doi: 10.1186/s12961-021-00778-y (PMC8665309; doi:10.1186/s12961-021-00778-y)
Supplement: Supplementary file 3 — Additional file 3. Description of Additional file 2 Tables S1–S6. [file 12961_2021_778_MOESM3_ESM.docx]

**Additional file 3**

**Description of Additional file 2 tables**

**Table S1** presents the bibliometric data collected in Scopus and SciVal. The total number of outputs (with at least one author from an African state) published between 2008 and 2017 ranged from 25 in São Tomé and Príncipe to 63,171 in South Africa. On a per-capita basis, the Seychelles had 3,568 publications per thousand population, while South Sudan had only 0.007 publications per thousand people. The absolute number of citations for published outputs ranged from 335 in São Tomé and Príncipe to 243,026 in Kenya.^[[1]](#footnote-1)^ Three quarters (43/54) of country publications included international authors, yet a small share of total publications was first authored by researchers based in each country.

**Table S2** shows the data on clinical trial infrastructures and intellectual property rights. The number of clinical trials indexed in the World Health Organization’s International Clinical Trials Registry Platform ranged from 0 in Cape Verde to 4,341 in South Africa. On a per capita basis, South Africa also had the highest number of registered trials (77.5 per million population) and some regional differences could be seen. In general Anglophone Eastern and Southern African nations appeared to have more trials per capita than many Francophone African countries, although Guinea Bissau and The Gambia stand out as having significantly more trials per capita to other surrounding countries. The number of patent applications by residents (2016, or last available year) ranged from 1 in Botswana, Djibouti and Tanzania to 2,783 in South Africa. Per capita, the highest number of patents filed South Africa, followed by Tunisia and Egypt – three of the higher income countries in the region. However, these were followed by Sudan as the fourth most prolific patent producer, despite a much lower income level.

**Tables S3** and S**4** present R&D personnel and spending, respectively. Based on data from 2016, or the nearest available year, the number of researchers per million inhabitants ranged from 7 in the Democratic Republic of Congo to 1,965 in Tunisia; the proportion of researchers with doctoral or equivalent degrees ranged from 10% in Malawi to 72% in Cape Verde. The gross expenditure on R&D as a share of GDP ranged from 0.01% in Madagascar to 0.8% in South Africa. The proportion of gross expenditure on R&D that went to the medical and health sciences ranged from 0% in Lesotho to 30% in Swaziland.

**Table S5** presents data on regulatory capacities. About half the countries had a national public health institute (27/54) and national ethics committee (25/54). The number of institutional review boards ranged from 0 in several countries to 30 in South Africa.

**Table S6** shows the amount of funding awarded to researchers in each country (2008-2017) from ten large organisations. These data indicated only 29 countries receiving funding from these organisations, apparently leaving 25 others without any research funded by the ten largest research funders. Some funding bodies spread resources widely across countries, such as the US National Institutes of Health, while others like the UK Medical Research Council appeared to focus on just a few countries (with more than half of its research expenditure in Africa going to the Gambia, according to data obtained).

1. No citation or first-author publication data were retrievable for South Africa or Egypt [↑](#footnote-ref-1)
